# MiR-4664-3p as a potential diagnostic, prognostic, and immunotherapeutic biomarker in NSCLC: modulation of tumor progression through CD8 + T cell regulation

**DOI:** 10.3389/fonc.2025.1642999

**Published:** 2025-11-04

**Authors:** Chun Yi, Hao Zhang, Qianqian Guo, Linzhu Lu, Cong Gao, Yunlong Zhao, Yan Su, Jing Lu

**Affiliations:** ^1^ Medical School, Hunan University of Chinese Medicine, Changsha, Hunan, China; ^2^ School of Integrated Traditional Chinese and Western Medicine, Hunan University of Chinese Medicine, Changsha, Hunan, China; ^3^ Institute of Ophthalmology and Otolaryngology of Chinese Medicine, Changsha, China; ^4^ Hunan Provincial Key Laboratory for the Prevention and Treatment of Ophthalmology and Otolaryngology Diseases with Chinese Medicine, Changsha, China

**Keywords:** NSCLC, PRKCB, miR-4664-3p, immune microenvironment, biomarker

## Abstract

**Introduction:**

Non-small cell lung cancer (NSCLC) is one of the leading causes of cancer-related deaths worldwide, largely due to complex interactions within the tumor-immune microenvironment that limit treatment efficacy. MicroRNAs (miRNAs) play critical roles in the regulation of tumor progression and immune evasion. This study systematically evaluated the expression characteristics, clinical significance, and role of miR-4664-3p in tumor immune regulation in NSCLC.

**Methods:**

We analyzed an NSCLC dataset from The Cancer Genome Atlas (TCGA) and identified miR-4664-3p as a potential diagnostic, prognostic, and immunotherapeutic biomarker. Bioinformatic approaches have been used to assess miRNA expression and clinical significance. The regulatory role of the miR-4664-3p/Protein Kinase C Beta (PRKCB) axis was further examined using correlation analysis, nomogram construction, and experimental validation in cell lines and animal models.

**Results:**

MiR-4664-3p was significantly upregulated in NSCLC tissues and served as an independent predictor of poor prognosis. Its increased expression was linked to reduced immune cell infiltration and enhanced immune escape. PRKCB was validated as a direct downstream target of miR-4664-3p and showed a positive association with CD8 + T cell infiltration and favorable outcomes. Functional assays confirmed that miR-4664-3p promoted NSCLC cell proliferation, migration, and invasion. Conversely, the inhibition of miR-4664-3p increased PRKCB expression, boosted CD8 + T cell activity, strengthened anti-tumor immunity, and suppressed tumor growth.

**Conclusion:**

These results suggest that the miR-4664-3p/PRKCB axis is crucial in NSCLC progression and immune modulation. Hence, MiR-4664-3p is a potential diagnostic and prognostic indicator, as well as therapeutic target in immunotherapy strategies for NSCLC.

## 1 Introduction

Non-small cell lung cancer (NSCLC) accounts for approximately85% of all lung cancer cases, making it the dominant clinicopathological subtype (1, 2). However, despite the development of targeted therapies, such as tyrosine kinase inhibitors (TKIs) and immunotherapies involving anti-PD-L1 monoclonal antibodies (mAbs), the prognosis for patients with NSCLC remains unfavorable, particularly for those diagnosed with advanced-stage disease (3–5).

The complex and evolving nature of the tumor microenvironment (TME) is key in the limited clinical efficacy observed in NSCLC. The TME is crucial for driving NSCLC progression, facilitating immune escape, and contributing to therapy resistance (6). Recently, research on TME-related biomarkers has progressed, and several molecules have been identified for disease diagnosis, prognostic evaluation, and prediction of treatment responses. For instance, PD-L1 expression levels are commonly employed to guide immune checkpoint inhibitor (ICI) therapy. Tumor mutational burden (TMB) serves as a predictor of immunotherapy efficacy, and the detection of driver gene mutations, including EGFR, ALK, and KRAS, has become essential for selecting targeted treatments (7–10). These biomarkers partially reflect the immune status within the TME and are reference points for clinical decision making. Nevertheless, their clinical applicability remains constrained by factors such as limited sample size, tissue heterogeneity, and uneven spatial distribution, which undermine the accuracy and generalizability of these markers. Recently, bioinformatic approaches have enabled the identification of biomarkers that are closely associated with tumorigenesis and disease progression using large-scale transcriptomic datasets. This advancements have facilitated improvements in diagnostic precision and prognostic evaluation systems for NSCLC (11). Thus, identifying novel molecular markers with diagnostic and immunological significance is crucial to fulfill the clinical need for early detection, improved prognostic stratification, and enhanced therapeutic response in NSCLC.

Studies have shown that certain miRNAs exert oncogenic effects in tumors by regulating processes such as cell proliferation, apoptosis, angiogenesis, metastasis, and immune modulation (12, 13), while some miRNAs exhibit tumor-suppressive functions and possess potential biomarker value in the diagnosis and prognosis of cancers (14). miRNAs are a class of small, non-coding RNAs of 18 to 25 nucleotides in length that regulate gene expression at the post-transcriptional level (15). They influence specific biological processes by binding to complementary sequences within the 3′ untranslated regions (3’ UTR) of target mRNAs, thus regulating gene expression (16). Numerous miRNAs exhibit altered expression patterns in NSCLC, making them potential candidates for diagnostic and prognostic biomarkers (17). In addition, miRNAs play important roles in shaping the tumor immune microenvironment by regulating immune cell infiltration, antigen presentation, and the expression of immune checkpoints. For example, the microRNA-183/96/182 cluster can promote the enrichment of CD8^+^ cytotoxic T cells within the tumor microenvironment by regulating the secretion of interleukin-2, thereby inhibiting the progression and metastasis of NSCLC (18). miR-873 and miR-105–2 are significantly upregulated in lung adenocarcinoma tissues, are associated with poorer patient survival outcomes, and may influence the infiltration of monocytes and dendritic cells through immune-related pathways (19). MiR-155 can directly target cytokine response elements to suppress the expression of the programmed cell death protein 1 (PD-1) gene and is considered a potential biomarker for evaluating prognosis and therapeutic response in NSCLC (20). Although many miRNAs have been linked to the TME in NSCLC, their clinical translation remains limited owing to insufficient mechanistic understanding and inadequate validation using large-scale datasets. In NSCLC, differentially expressed miRNAs may regulate target gene networks associated with immunity and tumor progression, thereby influencing the state of the tumor immune microenvironment and participating in disease progression as well as affecting patient prognosis.

Through comprehensive screening and multidimensional validation, we identified that miR-4664-3p was significantly upregulated in tumor tissues and closely associated with poor prognosis, suggesting that it may play an important role in the initiation and progression of NSCLC. Previous studies have shown that miR-4664-3p promotes tumor progression by directly targeting CDK2AP2, thereby regulating the cell cycle, and that its expression is associated with patient survival outcomes in certain cohorts (21). In studies of lung adenocarcinoma (LUAD), miR-4664-3p was included in a six-miRNA prognostic signature and found to correlate with patient survival; however, the direction of its effect remained unclear and mechanistic validation was lacking (22). In small-cell carcinoma of the esophagus (SCCE), miR-4664-3p was significantly associated with postoperative recurrence, but the original study did not provide directional information or hazard ratios, leaving its role as a prognostic biomarker to be further elucidated (23). However, existing research has mainly focused on the role of miR-4664-3p in cell proliferation and cell-cycle regulation, while its potential functions in immune regulation and the tumor immune microenvironment have not yet been fully clarified. Therefore, this study further hypothesizes that miR-4664-3p may influence the state of the tumor immune microenvironment by regulating immune-related target gene networks, thereby contributing to NSCLC progression and affecting patient prognosis. Based on data from The Cancer Genome Atlas (TCGA, https://portal.gdc.cancer.gov/) and supported by both *in vitro* and *in vivo* experiments, this study systematically evaluates the role of miR-4664-3p from three perspectives—expression characteristics, immune relevance, and potential mechanisms—with the aim of elucidating its biological function in NSCLC and its potential clinical value as a diagnostic, prognostic, and immunotherapy-related biomarker.

## 2 Materials and methods

### 2.1 Data acquisition and preprocessing

The data used in this study were obtained from TCGA, which contains comprehensive gene expression profiles, miRNA expression data, and clinical follow-up information for 33 cancer types. Specifically, data from NSCLC samples comprising 1,153 transcriptomic samples (110 normal and 1,043 tumor tissues), 1,090 miRNA samples (91 normal and 999 tumor tissues), and 1,026 clinical samples were extracted (**Supplementary Table 1**). Gene and miRNA expression matrices were downloaded in TSV format, and clinical information was obtained in BCR-XML format. The expression data were preprocessed and normalized using the R software (version 4.4.2), and mRNA data were extracted for subsequent analyses using the biotype.pl script. After formatting and handling of missing values, the clinical data were retained with variables including age, sex, TNM stage, survival status, and survival time.

### 2.2 Screening and prognostic analysis of differentially expressed miRNAs

Differential analysis of miRNA expression data between NSCLC tumor and normal tissues was performed using the “limma” package in R software (version 4.4.2). The selection criteria were set as |log_2_FC| > 1 and FDR < 0.05. The results of the differential expression analysis were used for subsequent visualization and survival-related evaluation. The differentially expressed miRNAs were then combined with patient survival data, and their associations with overall survival (OS) were assessed using Kaplan–Meier (K–M) survival analysis and univariate Cox regression models. In the survival analysis, the median expression level of each miRNA was used as the cutoff value to divide patients into high- and low-expression groups. miRNAs showing statistically significant associations in the univariate analysis were further evaluated by multivariate Cox regression to determine their independent prognostic value. Subsequently, time-dependent receiver operating characteristic (ROC) curve analysis was conducted to comprehensively evaluate the prognostic discrimination ability of the candidate miRNAs, and the area under the curve (AUC) was calculated. miRNAs that met both differential expression and prognostic significance criteria were included in the subsequent analyses.

### 2.3 Expression characteristics and clinical correlation analysis of miR-4664-3p

Based on the standardized miRNA expression data from The Cancer Genome Atlas (TCGA) database, the expression differences of miR-4664-3p across 33 cancer types and between NSCLC tumor and normal tissues were visualized using the “ggplot2” package in R software (version 4.4.2). In the NSCLC cohort, patients were divided into high- and low-expression groups according to the expression levels of miR-4664-3p. The K–M method combined with the log-rank test was applied to evaluate its effect on overall survival (OS). Incorporating relevant clinical variables, univariate and multivariate Cox regression analyses were performed to assess the independent prognostic value of miR-4664-3p. A nomogram model was then constructed based on a multivariate Cox proportional hazards regression model, integrating the expression level of miR-4664-3p and clinical variables that were statistically significant in the multivariate analysis. The model was built using the rms package in R software (version 4.4.2) to predict 1-, 3-, and 5-year survival probabilities. The concordance index (C-index) and calibration curves were calculated to evaluate the predictive performance of the model. Furthermore, to assess the potential diagnostic discriminatory ability of miR-4664-3p, a ROC curve analysis was performed using TCGA-NSCLC miRNA expression data. The standardized expression level of miR-4664-3p was used as the predictor variable, and the tissue type (tumor vs. normal) was set as the outcome variable. The area under the curve (AUC) and its 95% confidence interval (CI) were calculated, and the Youden index was used to determine the optimal cutoff value for evaluating the model’s sensitivity and specificity.

### 2.4 TIME and immunotherapy response analysis

Based on the transcriptomic expression data of patients with NSCLC from The Cancer Genome Atlas (TCGA) database, the Estimation of STromal and Immune cells in MAlignant Tumor tissues using Expression data (ESTIMATE) algorithm was used to calculate the StromalScore, ImmuneScore, and ESTIMATEScore. The analysis was conducted using the “estimate” package in R software (version 4.4.2), and the results were visualized using the “ggplot2” package. The potential for immunotherapy response was predicted using the Tumor Immune Dysfunction and Exclusion (TIDE) online platform (http://tide.dfci.harvard.edu/), with the standardized mRNA expression matrix (Transcripts Per Million, TPM) used as input. Samples were grouped according to miR-4664-3p expression levels, and the differences in TIDE scores between the high- and low-expression groups were compared.

### 2.5 Target gene prediction and functional annotation analysis

Potential target genes of miR-4664-3p were predicted using the TargetScan database (https://www.targetscan.org/). Differentially expressed genes (DEGs) in NSCLC were screened from the Gene Expression Profiling Interactive Analysis (GEPIA, http://gepia.cancer-pku.cn/) database, and the intersection of these two datasets was obtained to identify candidate genes. The candidate genes were imported into the Search Tool for the Retrieval of Interacting Genes/Proteins (STRING, https://string-db.org/) database to construct a protein–protein interaction (PPI) network, and the top 10 core genes were selected using the cytoHubba plugin in Cytoscape software based on the MCC algorithm. Subsequently, the candidate genes were subjected to Gene Ontology (GO, http://geneontology.org/) functional enrichment and Kyoto Encyclopedia of Genes and Genomes (KEGG, https://www.kegg.jp/) pathway enrichment analyses using the “clusterProfiler” package in R software (version 4.4.2), with a screening threshold of a corrected P-value < 0.05.

### 2.6 Expression characteristics and prognostic analysis of protein kinase C beta

Based on the Gene Expression Profiling Interactive Analysis (GEPIA, http://gepia.cancer-pku.cn/) database, the expression differences of core genes between NSCLC tumor and normal tissues were analyzed. K–M survival analysis and the log-rank test were subsequently performed to evaluate the relationship between core gene expression levels and overall survival (OS). By integrating the results of differential expression and survival analyses, candidate key genes with potential clinical significance were identified for subsequent miRNA target prediction and functional validation. Subsequently, the miRanda algorithm (version 3.3a) was used to align the 3′ untranslated region (3′UTR) sequence of miR-4664-3p with that of PRKCB to predict potential binding sites.

### 2.7 Correlation analysis

Pearson correlation analysis was performed to evaluate the expression correlation between miR-4664-3p and the candidate genes. The screening criteria were set as a correlation coefficient (r) < 0 and P < 0.05. A scatter plot was generated for the key gene PRKCB to visualize its co-expression relationship with miR-4664-3p.

### 2.8 ROC analysis

ROC curve analysis was performed using the pROC package in R software (version 4.4.2) to evaluate the diagnostic efficacy of PRKCB. The standardized expression level of PRKCB was used as the predictor variable, and the tissue category (tumor vs. normal) was used as the outcome variable. The area under the curve (AUC) and its 95% confidence interval (CI) were calculated, and the optimal cutoff value was determined based on the Youden index, thereby assessing the ability of PRKCB to distinguish between NSCLC tumor and normal tissues.

### 2.9 Analysis of the relationship between PRKCB expression and immune microenvironment infiltration

Single-cell RNA sequencing data for NSCLC were obtained from the Tumor Immune Single-Cell Hub 2 (TISCH2, http://tisch.comp-genomics.org/) database. The t-distributed stochastic neighbor embedding (t-SNE) algorithm was applied for dimensionality reduction and visualization of cell type distribution to analyze the expression characteristics of PRKCB across different immune cell subpopulations. Using immune cell marker gene sets, single-sample gene set enrichment analysis (ssGSEA) was performed with the GSVA package in R software (version 4.4.2) to estimate immune cell infiltration levels. Visualization was conducted using violin and box plots generated with the ggplot2 package. Furthermore, the correlation between PRKCB expression and immune cell infiltration was explored through Spearman’s correlation analysis using lung adenocarcinoma (LUAD) and lung squamous cell carcinoma (LUSC) samples from the Tumor Immune Estimation Resource 2.0 (TIMER2.0, http://timer.cistrome.org/) database.

### 2.10 Cell culture, grouping, and treatment

A549 (Hunan Fenghui Biotechnology Co., Ltd., catalog no. CL0024), NCI-H1975 (catalog no. CL0232), and Lewis cells (catalog no. CL0194) were cultured in high-glucose Dulbecco’s modified Eagle medium (DMEM; VivaCell, catalog no. PM150210) supplemented with 10% fetal bovine serum (FBS; Procell, catalog no. 164210) at 37 °C in a 5% CO_2_ atmosphere. When cells reached 60–70% confluency, transfection was conducted using Lipofectamine™ 3000 (Invitrogen, Thermo Fisher Scientific, catalog no. L3000-015), according to the manufacturer’s instructions. Transfections included miR-4664-3p mimics, inhibitors, and their respective negative controls (NC mimics and NC inhibitors; RiboBio) at a final concentration of 20 μmol/L. After 24 h, the medium was replaced with fresh DMEM containing 10% FBS. Total RNA was isolated 48 h post-transfection and miR-4664-3p expression levels were quantified by real-time qPCR using the Bulge-Loop miRNA qRT-PCR Starter Kit (RiboBio, catalog no. C10211-2). U6 small nuclear RNA was used as the internal reference, and relative expression was calculated using the 2^^-^ΔΔCt^ method, the primer sequences are shown in the table (**Supplementary Table 2**).

### 2.11 CCK-8 assay

A549 and NCI-H1975 cells were seeded in 96-well plates at a density of 5 × 10 cells/well. Transfection was performed after the attachment of cells to the plate surface. At 24, 48, and 72 h post-transfection, 100 μL of medium containing 10% CCK-8 working solution (Biosharp, Cat No: BS350B) was added to each well. The cells were then incubated for 2 h at 37 °C in a 5% CO_2_ atmosphere. Absorbance was measured at 450 nm using a microplate reader. Each assay was performed in triplicate and the mean absorbance value from three replicates was used to calculate the cell proliferation inhibition rate (%).

### 2.12 5-Ethynyl-2’-deoxyuridine cell proliferation assay

At 48 h post-transfection, the EdU Cell Proliferation Imaging Assay Kit (Elabscience, Batch No: AK17802) was used in accordance with the manufacturer’s instructions. An EdU working solution (diluted 1:1000) was added to each group, followed by incubation at 37 °C for 2 hours. Subsequently, the cells underwent fixation, permeabilization, the E-click reaction, and nuclear staining with DAPI. Fluorescence images were captured using a fluorescence microscope and the proportion of EdU-positive cells was quantified using ImageJ software to assess DNA synthesis activity.

### 2.13 Cell scratch assay

A549 and NCI-H1975 cells were plated in 12-well plates at 3 × 10^5^ and 5 × 10^5^ cells/well, respectively. When cells reached confluence, a scratch was made using a sterile pipette tip. After rinsing with PBS, the medium was replaced with serum-free DMEM. Images of the wound were captured at 0, 24, and 48 h, and the wound width was measured using the ImageJ software to calculate the healing rate. All experiments were conducted in triplicates.

### 2.14 Transwell invasion assay

Transwell chambers (Costar, Cat No: 3422) precoated with Matrigel matrix (Corning, USA, Lot No: 2342002) were used to evaluate cell invasion. At 48 h post-transfection, A549 (2 × 10^4^ cells in 300 μL) and NCI-H1975 (4 × 10^4^ cells in 300 μL) cells were resuspended in serum-free DMEM and seeded into the upper chambers. The lower chambers were filled with DMEM supplemented with 10% fetal bovine serum (FBS), which served as a chemoattractant. Following 24 h of incubation at 37 °C in a 5% CO_2_ environment, cells were washed twice with PBS, fixed with 4% paraformaldehyde for 20 min, and stained using 0.1% crystal violet (Solarbio, Cat No: G1061) for 20 min. Images were acquired under an inverted microscope, and the number of invaded cells was quantified using ImageJ software.

### 2.15 Xenograft tumor model

Twelve male C57BL/6J mice (4–6 weeks old; Slaughter Kingda Laboratory Animal Co., Ltd., Hunan, China; License No. SCXK (Xiang) 2021-0002) were housed under standard conditions. Lewis lung carcinoma cells (1 × 10^6^) were injected into the right axilla. Seven days post-inoculation, the mice were assigned to two groups (n = 6 per group) and received intratumoral injections of miR-4664-3p antagomir or negative control (NC; RiboBio, Guangzhou) at 20 nmol in 20 μL PBS, every three days for four cycles. After treatment, RNA was isolated from the tumors. cDNA synthesis was performed using NovoScript^®^ Plus All-in-one 1st Strand cDNA Synthesis SuperMix (Novoprotein, Cat No: E047), and *PRKCB* expression was quantified using qRT-PCR with NovoStart^®^ SYBR SuperMix Plus (Novoprotein, Cat No: E096). β-actin served as the internal reference, and relative expression was calculated via the 2^^-^ΔΔCt^ method, the primer sequences are shown in the table(**Supplementary Table 3**). Tumor tissues were processed for immunofluorescence with antibodies against CD8 (Abcam, ab217344) and IFN-γ (Bioss, bs-0480R), using TSA reagent (Pino-Bio) for signal amplification. Fluorescence images were captured, and CD8 + cell count and IFN-γ intensity were measured with ImageJ.

### 2.16 Data statistics

All statistical analyses were performed using R software (version 4.4.2). K–M survival analysis combined with the log-rank test was applied to compare overall survival (OS) differences between groups. The Cox proportional hazards regression model was used to evaluate the effects of gene expression and clinical variables on OS, with results presented as hazard ratios (HRs) and their 95% confidence intervals (CIs). Survival predictive performance was assessed using time-dependent ROC curve analysis, while diagnostic discrimination ability was evaluated using ROC curve analysis. Pearson’s correlation coefficient was used to examine correlations. Categorical variables were compared using the chi-square test, and continuous variables were analyzed using Student’s t-test under the assumptions of normality and homogeneity of variance; otherwise, the Wilcoxon rank-sum test was applied. A P value < 0.05 was considered statistically significant.

## 3 Results

### 3.1 MiR-4664-3p is identified as a potential independent prognostic biomarker in NSCLC

**Figure 1:**
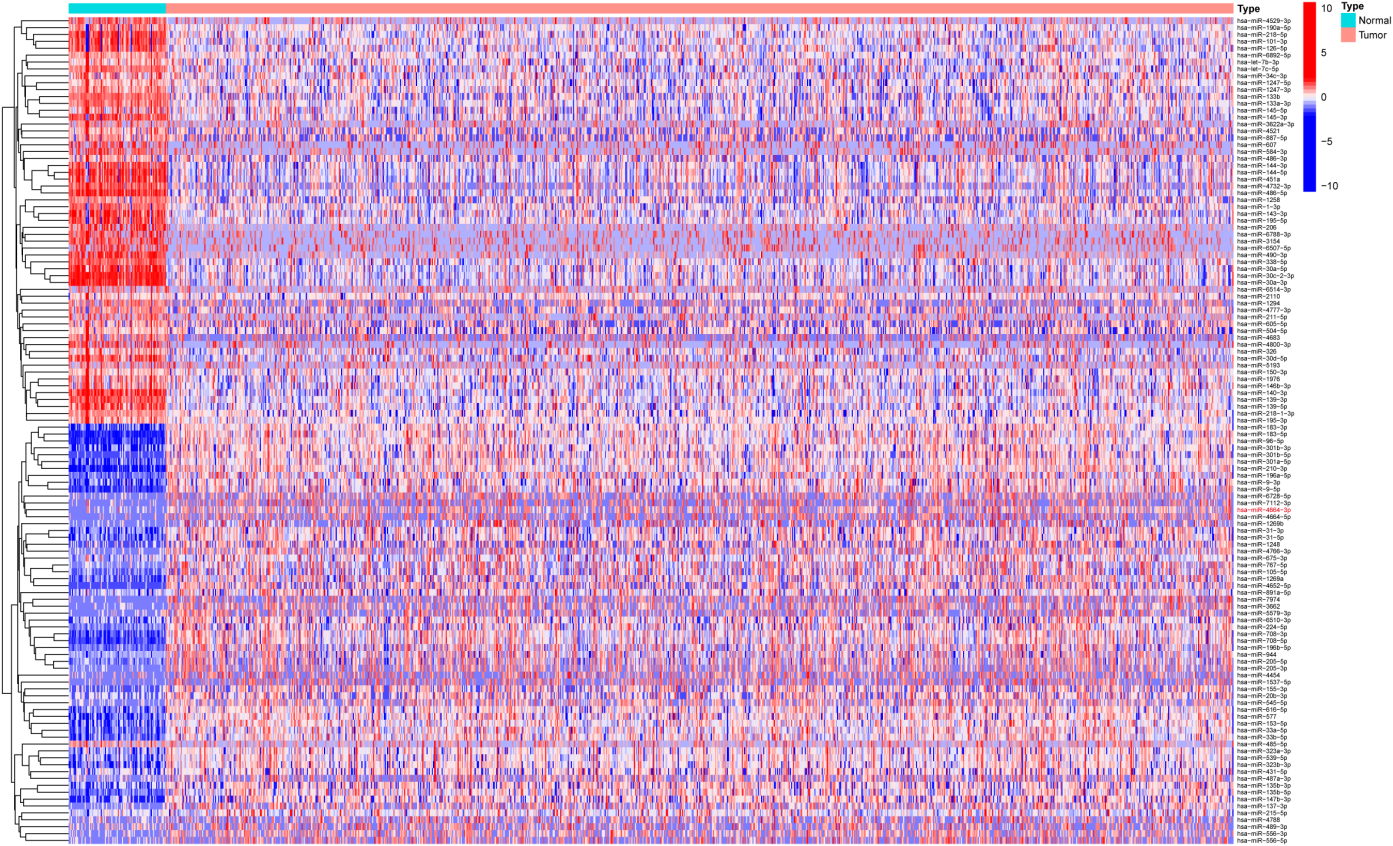
Heatmap of the top 50 upregulated and 50 downregulated miRNAs in NSCLC samples.

Based on the miRNA expression data of tumor and normal tissues from patients with NSCLC in the The Cancer Genome Atlas (TCGA) database, a total of 337 differentially expressed miRNAs were identified, including 269 upregulated and 68 downregulated in tumor tissues (**Supplementary Table 4**). To visually depict the differential expression patterns, the heatmap presents the top 50 upregulated and 50 downregulated miRNAs (**Figure 1**). These differentially expressed miRNAs were further analyzed in combination with patients’ clinical follow-up information to evaluate the association between miRNA expression levels and overall survival (OS). K–M survival analysis and univariate Cox regression identified 10 miRNAs significantly associated with patient prognosis (**Table 1**). In subsequent multivariate Cox regression analysis, after adjusting for variables such as age, sex, and clinical stage, five miRNAs with independent prognostic value were identified (**Table 2**). The ROC curve analysis was then performed to assess the survival predictive performance of these candidate miRNAs. The results indicated that miR-4664-3p exhibited relatively superior predictive performance among the candidates and was therefore selected for further analyses (**Supplementary Table 5**).

**Table 1:** miRNAs associated with survival identified by Kaplan–Meier and Cox regression analyses in NSCLC.

miRNA	HR	HR.95L	HR.95H	KMpvalue	COXpvalue
hsa-miR-187-3p	0.99857	0.997193	0.999948	0.014803	0.041987
hsa-miR-584-3p	1.264151	1.11916	1.427926	0.003621	0.000162
hsa-miR-101-3p	0.999979	0.999961	0.999997	0.032147	0.024274
hsa-miR-4664-3p	1.155921	1.056226	1.265025	0.005413	0.00164
hsa-miR-1910-5p	1.06849	1.002473	1.138853	0.04843	0.041763
hsa-miR-548v	0.9496	0.910533	0.990343	0.001396	0.015836
hsa-miR-195-3p	0.93987	0.891335	0.991048	0.006558	0.021884
hsa-miR-548d-3p	1.237743	1.03781	1.476193	0.017186	0.017652
hsa-miR-556-3p	0.836845	0.72003	0.972612	0.002918	0.020234
hsa-miR-195-5p	0.99214	0.986749	0.997561	0.014878	0.004537

**Table 2:** Independent prognostic miRNAs identified by multivariate Cox regression analysis in NSCLC.

Id	HR	HR.95L	HR.95H	Pvalue
hsa-miR-4664-3p	1.172252	1.070688	1.283449	0.000588
hsa-miR-548v	0.959778	0.922446	0.998621	0.042547
hsa-miR-548d-3p	1.265451	1.075576	1.488846	0.004535
hsa-miR-556-3p	0.840099	0.720105	0.980087	0.026709
hsa-miR-195-5p	0.993486	0.988027	0.998976	0.020095

### 3.2 Expression characteristics and clinical prognostic significance of miR-4664-3p in NSCLC

**Figure 2:**
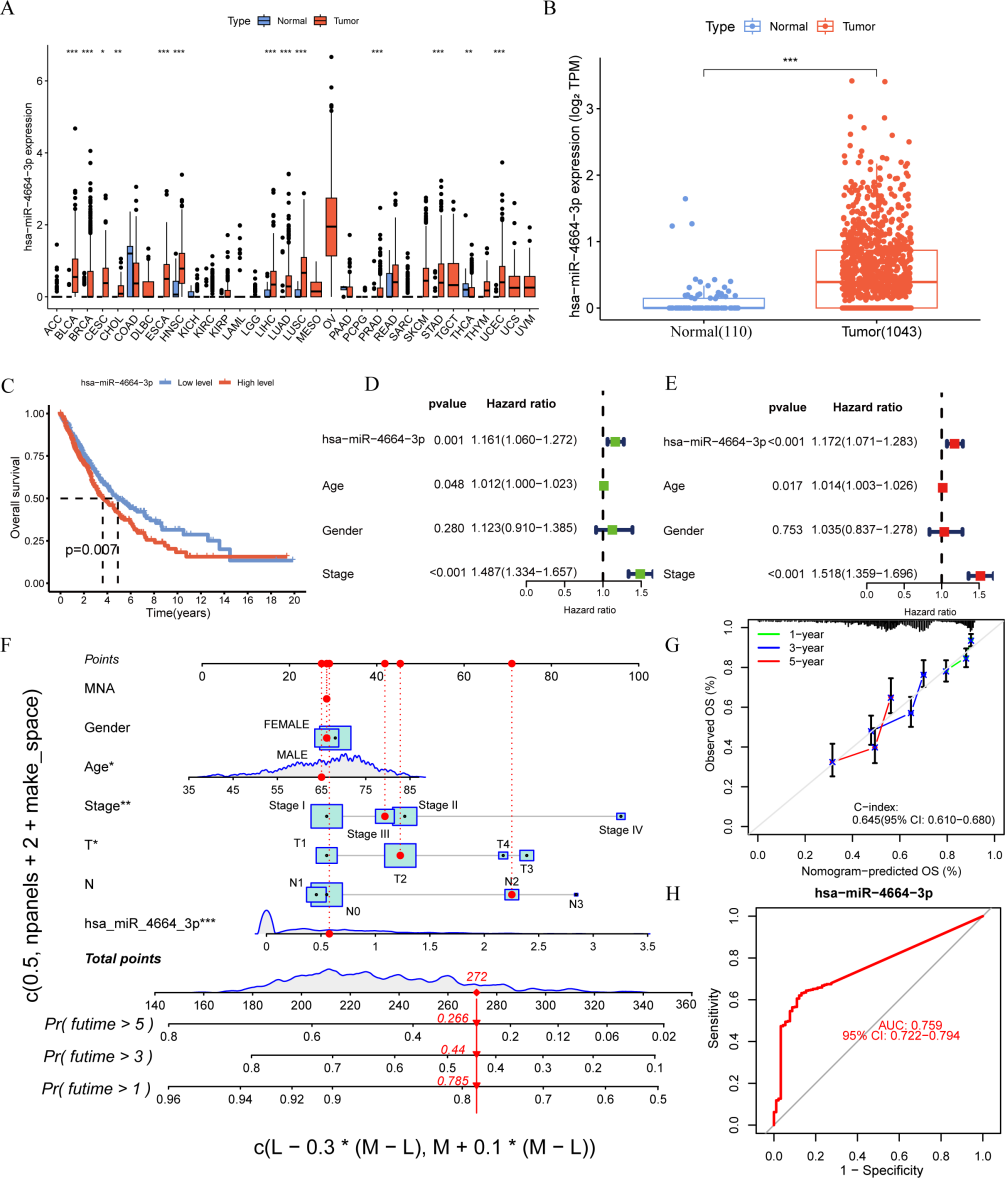
miR-4664-3p is highly expressed in NSCLC and has prognostic and diagnostic value. **(A)** Expression levels of miR-4664-3p across 33 human cancer types were analyzed based on data from The Cancer Genome Atlas (TCGA) database (expression data for GBM were not available). The X-axis represents different cancer types (TCGA). **(B)** Differential expression analysis of miR-4664-3p between NSCLC tumor tissues (n = 1,043) and normal lung tissues (n = 110) in the TCGA database. **(C)** K-M survival curves showing the association between miR-4664-3p expression levels and OS in NSCLC patients. **(D, E)** Univariate and multivariate Cox regression analyses assessing the prognostic value of miR-4664-3p and clinical parameters. **(F)** Nomogram constructed using miR-4664-3p expression and clinical characteristics to predict 1-, 3-, and 5-year survival probabilities in NSCLC patients. **(G)** Calibration curves evaluating the predictive accuracy of the nomogram model at 1, 3, and 5 years; the concordance index(C-index) indicates the model’s discriminatory ability. **(H)** ROC curve analysis of miR-4664-3p for diagnostic performance in distinguishing NSCLC tumor tissues from normal tissues. *P < 0.05, **P < 0.01, ***P < 0.001. ns, not significant. Abbreviation: TCGA, The Cancer Genome Atlas.

To thoroughly evaluate the expression profile of miR-4664-3p across various cancers, a pan-cancer study was performed using TCGA data. These findings revealed that miR-4664-3p was overexpressed in multiple human cancers, indicating its potential involvement in the pathogenesis of various malignancies (**Figure 2A**). Subsequent analysis of miRNA expression profiles comparing NSCLC normal lung tissues revealed a marked upregulation of miR-4664-3p in NSCLC specimens, indicating its potential oncogenic role in NSCLC (**Figure 2B**). To assess the prognostic relevance of miR-4664-3p, we analyzed its expression in relation to overall survival (OS), using the median expression level of miR-4664-3p as the cutoff. Kaplan–Meier survival curves indicated that the OS of the high-expression group was significantly lower than that of the low-expression group, suggesting that high miR-4664-3p expression is associated with poor prognosis and has potential as a prognostic biomarker (**Figure 2C**). Cox regression analysis was used to examine its independent prognostic value. Univariate Cox analysis revealed a significant correlation between increased miR-4664-3p levels and unfavorable survival (hazard ratio [HR] = 1.161; 95% CI: 1.060–1.272; *P* = 0.001). Multivariate analysis, accounting for age, sex, and clinical stage, reinforced miR-4664-3p as an independent predictor of prognosis (HR = 1.172; 95% CI: 1.071–1.283; *P* < 0.001) (**Figures 2D–E**). Based on these results, a nomogram incorporating miR-4664-3p expression with clinical factors (age, sex, clinical stage, T/N stage, and MYCN amplification status) was constructed to estimate the 1-, 3-, and 5-year survival rates of patients with NSCLC. Every variable received a corresponding score on the “Points” scale of the nomogram, with the cumulative score reflecting personalized survival predictions. The estimated 1-, 3-, and 5-year survival rates were 0.785, 0.440, and 0.266, respectively, indicating the reliability of the model for prognostic assessment (**Figure 2F**). The nomogram exhibited a concordance index (C-index) of 0.645 (95% CI: 0.610–0.680), indicating moderate discriminative ability. Calibration plots demonstrated a high degree of concordance between the predicted and observed survival rates at 1, 3, and 5 years (**Figure 2G**). Additionally, ROC curve analysis revealed that miR-4664-3p effectively distinguished NSCLC tumor from normal tissues, with an area under the curve (AUC) of 0.759 (95% CI: 0.722-0.794) (**Figure 2H**). In summary, high miR-4664-3p expression is not only as an independent predictor of poor prognosis in NSCLC, but also exhibits robust diagnostic value. The nomogram combining clinical features with miR-4664-3p expression demonstrated strong performance in survival prediction.

### 3.3 MiR-4664-3p shapes the immune microenvironment and correlates with immune escape in NSCLC

**Figure 3:**
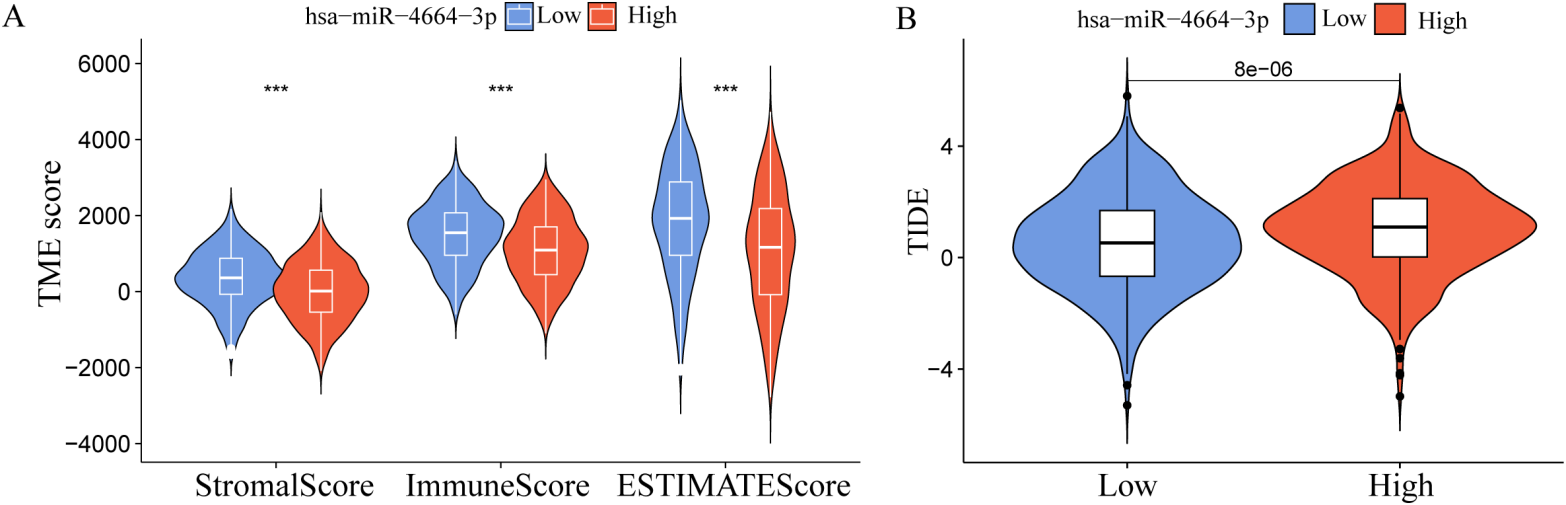
miR-4664-3p in the immune microenvironment of NSCLC and its association with immune escape. **(A)** Comparison of Tumor microenvironment (TME) scores between high and low miR-4664-3p expression groups using the ESTIMATE algorithm. **(B)** Evaluation of immune escape potential between high and low miR-4664-3p expression groups using the Tumor Immune Dysfunction and Exclusion (TIDE) algorithm. ***P < 0.001.

To explore the possible involvement of miR-4664-3p in the TME of NSCLC, differences in TME-related scores across the high- and low-expression groups were assessed using the ESTIMATE algorithm (**Figure 3A**). The analysis revealed that StromalScore, ImmuneScore, and ESTIMATEScore were significantly lower in the high miR-4664-3p expression group than in the low-expression group, indicating that increased miR-4664-3p expression may be associated with reduced infiltration of immune and stromal cells within the TME. To further investigate the relationship between miR-4664-3p and immune evasion, the Tumor Immune Dysfunction and Exclusion (TIDE) platform was used (**Figure 3B**). The analysis revealed a notable increase in TIDE scores in the high-expression cohort, suggesting that elevated miR-4664-3p levels are associated with enhanced immune evasion and decreased responsiveness to immunotherapy.

### 3.4 PRKCB is recognized as a direct target of miR-4664-3p and a potential diagnostic biomarker in NSCLC

**Figure 4:**
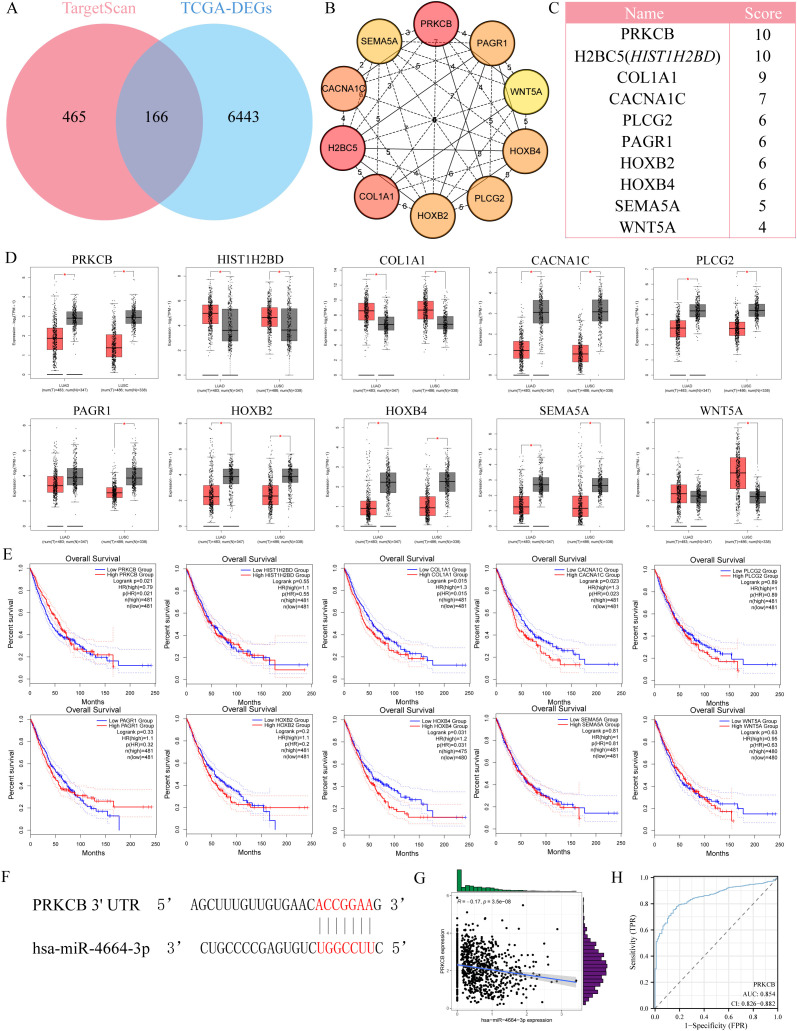
Identification of *PRKCB* as a target gene of miR-4664-3p in NSCLC. **(A)** Venn diagram showing the overlap between predicted target genes of miR-4664-3p and differentially expressed genes (DEGs) in NSCLC. **(B)** Protein*-*protein interaction (PPI) network of the top 10 hub genes identified using the cytoHubba plugin in Cytoscape. **(C)** Ranking of the top 10 hub genes based on MCC scores. **(D)** Expression levels of hub genes in NSCLC tumor tissues versus normal lung tissues based on TCGA data. **(E)** K-M survival analysis evaluating the association between hub gene expression and OS of NSCLC patients. **(F)** Predicted binding site between miR-4664-3p and the 3′ untranslated region (3′ UTR) of *PRKCB* identified by the miRanda algorithm. **(G)** Scatter plot showing Pearson correlation between miR-4664-3p and *PRKCB* expression levels. **(H)** ROC curve analysis evaluating the diagnostic performance of *PRKCB* in distinguishing NSCLC tumor tissues from normal tissues. *P < 0.05 ns: not significant.

To identify the potential target genes of miR-4664-3p in non-small cell lung cancer (NSCLC), we integrated differential gene expression data with target gene prediction results, leading to the identification of 166 candidate genes (**Figure 4A**). GO enrichment analysis revealed that these genes are significantly involved in biological processes such as T cell activation and interferon-γ production. KEGG pathway analysis showed their major enrichment in immune response pathways, including the T cell receptor signaling pathway and the JAK-STAT signaling pathway (Supplementary **Figure 1** A-B), suggesting that miR-4664-3p may regulate immune-related targets to participate in tumor immune modulation. Further screening led to the identification of 10 core genes (**Figures 4B, C**). Among them, only PRKCB was significantly downregulated in NSCLC tissues, and its high expression was significantly associated with better prognosis (**Figures 4D, E**), thus it was identified as the key target gene of miR-4664-3p. Sequence pairing using the miRanda v3.3a algorithm revealed a potential binding site for miR-4664-3p in the 3′ UTR region of PRKCB (**Figure 4F**). Further Pearson correlation analysis (**Figure 4G**) indicated a negative correlation between their expression levels (R = –0.17, p = 3.5e–08). ROC analysis showed that the AUC of PRKCB in distinguishing NSCLC tumor tissues from normal tissues was 0.854 (95% CI: 0.826–0.882), demonstrating good diagnostic performance (**Figure 4H**).

### 3.5 Increased PRKCB expression is associated with increased infiltration of immune effector cells in the TIME

**Figure 5:**
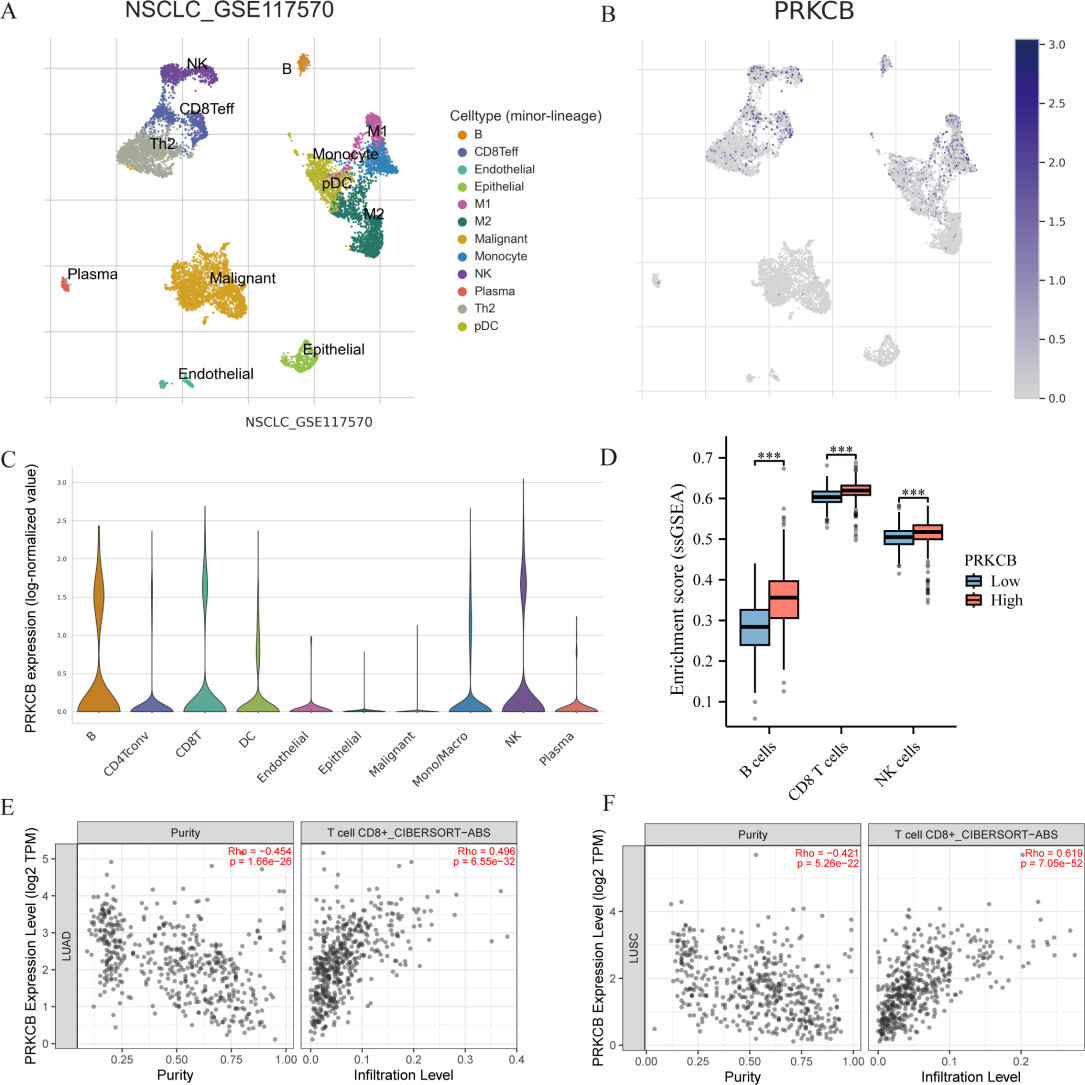
Correlation between *PRKCB* expression and immune cell infiltration in NSCLC. **(A)** Uniform manifold approximation and projection (UMAP) plot showing the distribution of distinct cellular subpopulations in NSCLC based on single-cell transcriptome data (GSE117570). **(B)** UMAP plot displaying *PRKCB* expression across different cell types. **(C)** Violin plot showing PRKCB expression levels in various immune cell types. The X-axis represents different cell types. **(D)** Single-sample gene set enrichment analysis (ssGSEA) of immune cell infiltration scores between high and low PRKCB expression groups.The X-axis represents different immune cell types. **(E, F)** TIMER 2.0 analysis showing the correlation between *PRKCB* expression and CD8^+^ T cell infiltration levels in lung adenocarcinoma(LUAD) and lung squamous cell carcinoma (LUSC). ***P < 0.001.

Analysis of single-cell transcriptomic data from the TISCH2 database (GSE117570) revealed that PRKCB is highly expressed in B cells, CD8^+^ effector T cells (CD8 Teff), and NK cells, while its expression is low in malignant tumor cells (**Figures 5A–C**). Grouping based on PRKCB expression levels showed that in the high PRKCB expression group, the infiltration levels of B cells, CD8^+^ T cells, and NK cells were significantly increased (**Figure 5D**). Further analysis using the TIMER2.0 database confirmed that in lung adenocarcinoma (LUAD) and lung squamous cell carcinoma (LUSC), PRKCB expression was positively correlated with the infiltration of the aforementioned immune cells, with the strongest correlation observed with CD8^+^ T cells (R = 0.325, P < 0.001) (**Figures 5E, F**; **Supplementary Figures 2A–D**). Taken together, these findings suggest that PRKCB may play an active role in the immune microenvironment of NSCLC by enhancing the infiltration and activity of immune effector cells such as CD8^+^ T cells.

### 3.6 MiR-4664-3p enhances NSCLC cell proliferation, migration, and invasion

**Figure 6:**
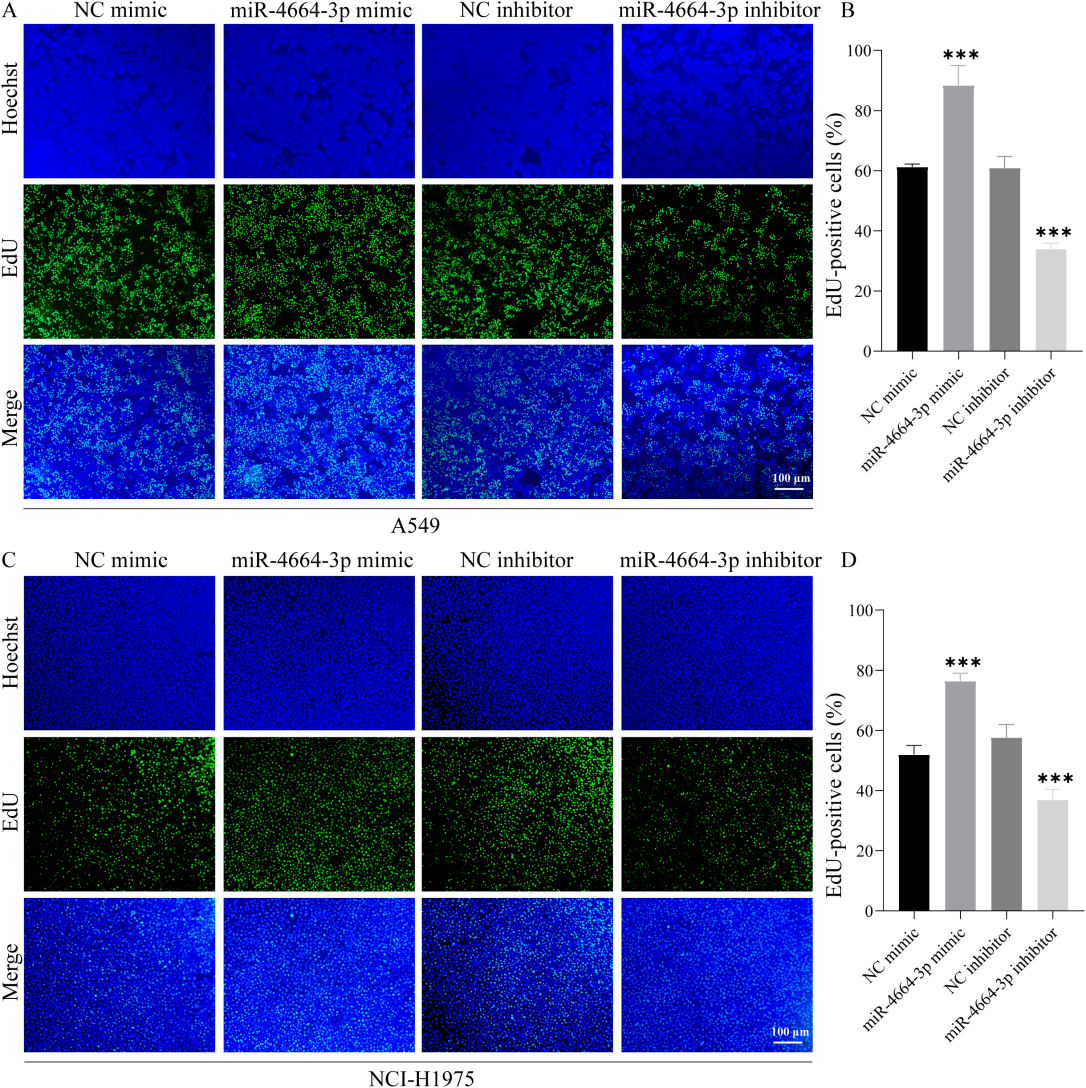
miR-4664-3p promotes NSCLC cell proliferation *in vitro*. **(A, C)** EdU staining images: nuclei labeled with Hoechst (blue) and proliferating cells labeled with EdU (green). **(B, D)** Quantitative analysis of the proportion of EdU-positive cells in each group. ***P < 0.001.

**Figure 7:**
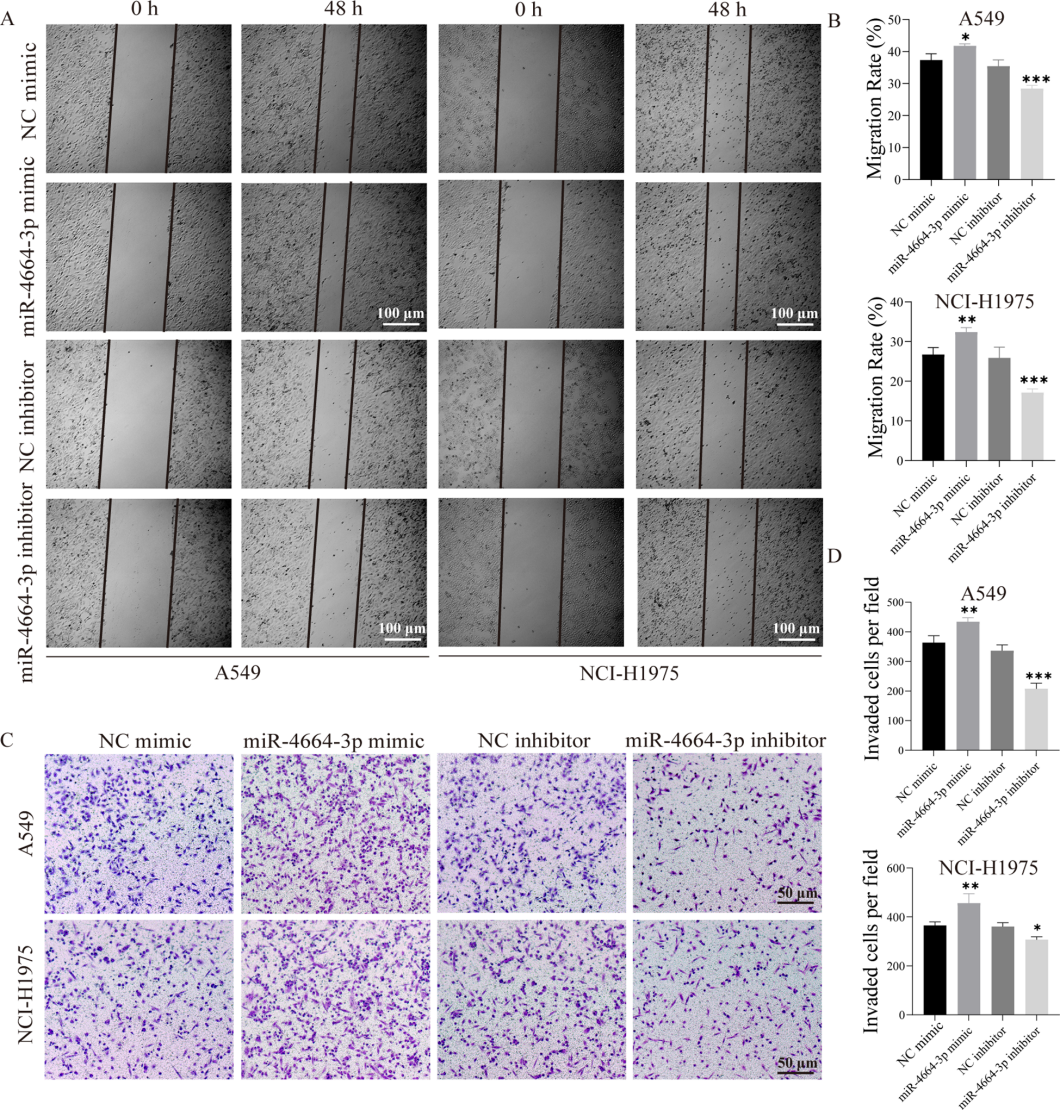
miR-4664-3p promotes NSCLC cell migration and invasion *in vitro*. **(A)** Scratch assay showing cell migration at 0 h and 48(h) **(B)** Quantitative analysis of migration rates in each group. The X-axis represents different treatment groups. **(C)** Transwell assay assessing cell invasion. **(D)** Statistical analysis of the number of invaded cells in each group. The X-axis represents different treatment groups. *P < 0.05, **P < 0.01, ***P < 0.001. ns, not significant.

To validate the functional role of miR-4664-3p in NSCLC cells, overexpression and inhibition experiments were conducted using miR-4664-3p mimics and inhibitors, respectively, in A549 and NCI-H1975 cell lines. The transfection efficiency was confirmed in both cell lines (**Supplementary Figures 3A–D**). Cell viability was evaluated at 0, 24, 48, and 72 h post-transfection using a CCK-8 assay. The results revealed that miR-4664-3p overexpression significantly increased the OD values relative to those of the control group, indicating enhanced cellular viability. Conversely, miR-4664-3p inhibition reduced OD values and suppressed cell viability (**Supplementary Figure 4A, B**). Consistently, EdU incorporation assays demonstrated an increased proportion of EdU-positive cells in the miR-4664-3p mimic group and a decreased proportion in that of the inhibitor group, confirming the proliferative effect of miR-4664-3p on NSCLC cells (**Figures 6A–D**). The cell migration capacity was assessed using a wound healing (scratch) assay. After 48 h of miR-4664-3p overexpression, the migration rate significantly exceeded that of the control group, whereas miR-4664-3p inhibition notably reduced the migration distance (**Figures 7A, B**). Additionally, Transwell invasion assays revealed that miR-4664-3p overexpression substantially promoted the invasive potential of NSCLC cells, whereas its inhibition suppressed invasion (**Figures 7C–D**). Collectively, these findings indicate that miR-4664-3p markedly enhances the proliferation, migration, and invasion capabilities of NSCLC cells.

### 3.7 Inhibition of miR-4664-3p suppresses tumor growth and enhances CD8^+^ T cell-mediated immune activation

**Figure 8:**
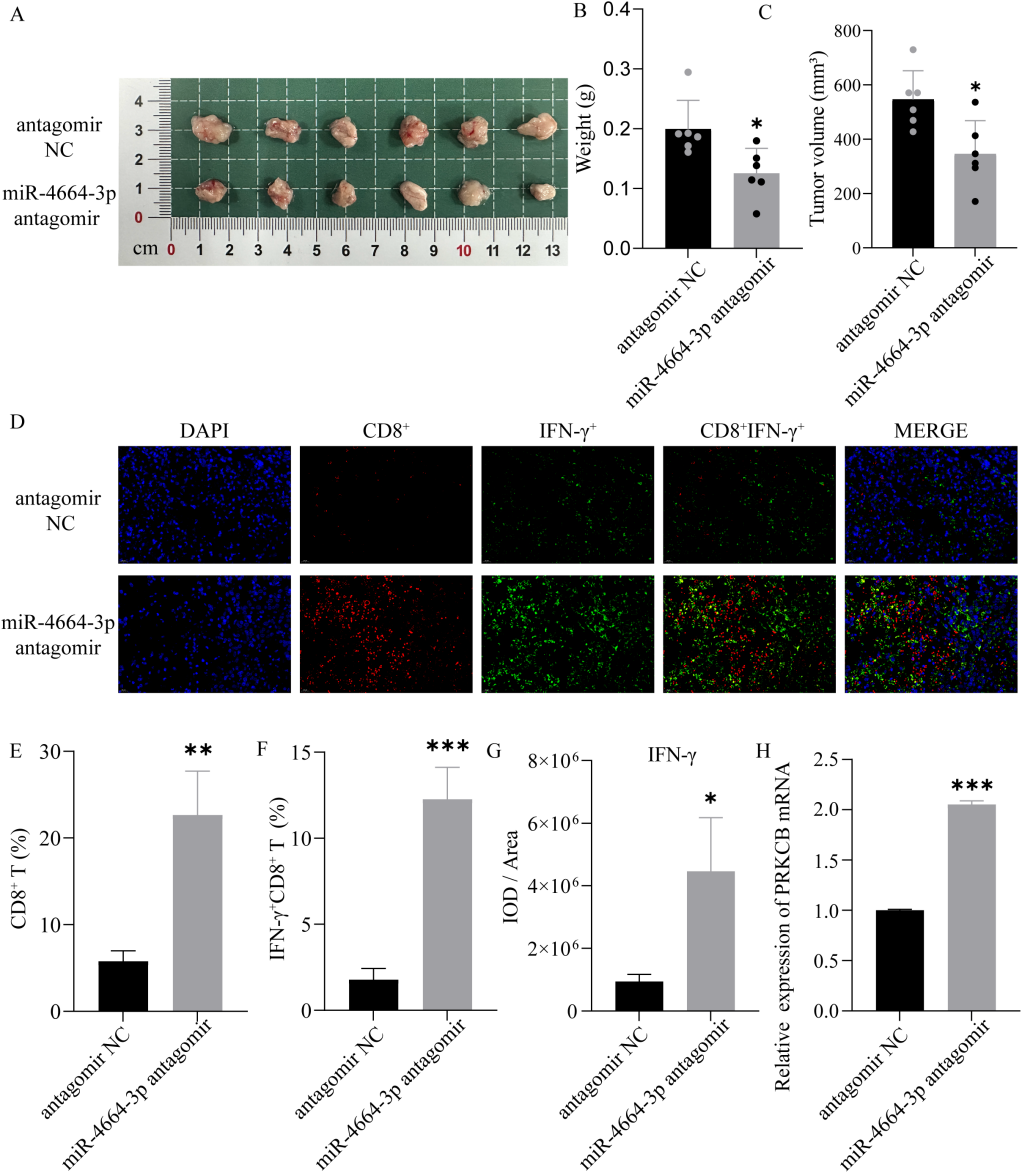
Role of miR-4664-3p antagomir in a NSCLC xenograft mouse model. **(A)** Gross appearance of tumors in each group. **(B)** Measurement of tumor weight. **(C)** Tumor volume comparison between antagomir NC and miR-4664-3p antagomir groups. **(D)** Immunofluorescence staining of CD8^+^ T cells (red) and IFN-γ (green) in tumor tissues; nuclei were counterstained with DAPI (blue). **(E, F)** Quantification of CD8⁺ T cells and IFNγ⁺ CD8⁺ T cells. **(G)** Measurement of IFN-γ fluorescence intensity (IOD/Area). **(H)** qRT-PCR analysis of PRKCB mRNA expression in tumor tissues. *P < 0.05, **P < 0.01, ***P < 0.001. ns, not significant.

To assess the *in vivo* role of miR-4664-3p in NSCLC progression, a subcutaneous xenograft tumor model was established in C57BL/6 mice. Upon tumor formation, the mice were administered intratumoral injections of either the miR-4664-3p antagomir or negative control antagomir (antagomir NC). The findings demonstrated that tumor volume and weight were notably diminished in the miR-4664-3p antagomir-treated group compared to those in the controls (**Figures 8A–C**). qRT-PCR analysis revealed that miR-4664-3p inhibition significantly upregulated *PRKCB* mRNA expression in tumor tissues (**Figure 8H**). Furthermore, immunofluorescence staining confirmed that suppression of miR-4664-3p resulted in an increased proportion of CD8 + T cells and IFN-γ + CD8 + T cells, accompanied by increased IFN-γ expression levels. These observations indicated that the inhibition of miR-4664-3p enhanced CD8 + T cell activation (**Figures 8D–G**).

## 4 Discussion

This study integrated transcriptomic and clinical data from the TCGA NSCLC cohort to systematically evaluate the clinical significance and biological role of miR-4664-3p. The results revealed that miR-4664-3p was significantly upregulated in NSCLC tissues, and its high expression was closely associated with poor survival outcomes. In multivariate Cox regression analysis, miR-4664-3p emerged as an independent unfavorable prognostic factor. The nomogram constructed based on miR-4664-3p and clinical variables demonstrated good discrimination and calibration, suggesting its potential as a prognostic evaluation tool. Diagnostic analysis also indicated that miR-4664-3p has some discriminatory ability between tumor and normal tissues. Given the stability of miRNAs in bodily fluids and the advantages of non-invasive detection (24), miR-4664-3p may serve as a potential biomarker for assisting clinical risk assessment, although its specific application value requires further investigation.

Previous studies have reported the tumor-related roles of miR-4664-3p from various perspectives. Liu et al. observed an upregulation of miR-4664-3p in NSCLC and demonstrated that it promotes cell proliferation and migration through the miR-4664-3p/CDK2AP2 axis (21). Li et al. included miR-4664-3p in a six-miRNA prognostic model for lung adenocarcinoma and proved its correlation with overall survival (22). In esophageal small cell carcinoma, miR-4664-3p was associated with postoperative recurrence (23). Although existing studies have shown that miR-4664-3p primarily acts as an oncogenic miRNA in NSCLC, its function in other tumor types remains unclear. It may exhibit a dual role, and the specific mechanisms require further exploration.

NSCLC, as a highly immunologically driven malignant tumor, involves immune cells, cytokines, and immune checkpoint molecules in its tumor microenvironment, all of which contribute to immune evasion, progression, and metastasis (25). Tumor cells often achieve immune escape by upregulating PD-L1, which binds to PD-1 on the surface of T cells, inducing T cell exhaustion (26). Immune checkpoint inhibitors (ICIs) such as PD-1/PD-L1 inhibitors have significantly improved survival in some NSCLC patients (27, 28). However, the differences in efficacy suggest that the complexity of the tumor immune microenvironment is one of the key factors affecting immune therapy responses. Our analysis found that high expression of miR-4664-3p was associated with decreased immune infiltration scores, suggesting that it may promote an immunosuppressive state. TIDE analysis further supports the association of miR-4664-3p with immune escape tendencies. *In vivo* experiments further demonstrated that inhibition of miR-4664-3p enhances CD8^+^ T cell infiltration and IFN-γ expression in tumor tissues, suggesting that miR-4664-3p may promote tumor progression by negatively regulating CD8^+^ T cell activity.

The composition and functional state of the TIME are pivotal in influencing the clinical outcomes of NSCLC patients. For instance, immunologically “hot” tumors, which are marked by increased T-cell infiltration and expression of inflammatory factors, generally show better responses to immunotherapy and more favorable prognoses. In contrast, immunologically “cold” tumors, with low T-cell infiltration and increased immunosuppression, are generally associated with poorer outcomes (29, 30). The TME of NSCLC comprises diverse immune cell populations, including CD8 + cytotoxic T cells, CD4 + helper T cells, Tregs, tumor-associated macrophages (TAMs), and dendritic cells (DCs) (31). Among these, CD8 + T cells serve as the primary effector cells responsible for tumor cell recognition and elimination, with their infiltration level and functional status directly influencing immunotherapy efficacy and prognosis (32). In many NSCLC cases, CD8 + T cells are reduced in number or functionally impaired, representing a hallmark of immune evasion (33, 34). Our analysis, using the ESTIMATE algorithm, revealed an inverse relationship between miR-4664-3p expression and immune cell infiltration. Consistent with this, TIDE analysis showed that increased expression of miR-4664-3p was associated with immune escape and a reduced response to immunotherapy, indicating its potential role in influencing the effectiveness of immune checkpoint inhibition. Notably, *in vivo* experiments revealed that inhibition of miR-4664-3p enhanced CD8^+^ T-cell infiltration within tumor tissues and increased IFN-γ expression levels. Upon activation by tumor-associated antigens, CD8 + T cells release multiple effector molecules, with IFN-γ being a prominent cytokine. IFN-γ promotes the secretion of perforin and granzymes, enhancing CD8 + T-cell cytotoxicity, and also induces chemokine secretion, including CXCL9 and CXCL10, which facilitate CD8 + T-cell infiltration into the TME, thereby amplifying the local immune response (35, 36). Our study confirmed that inhibiting miR-4664-3p significantly enhanced the infiltration of CD8 + T cells and the release of IFN-γ in tumor tissues, leading to a stronger anti-tumor immune response and suppression of tumor growth. These findings elucidated the potential mechanism by which miR-4664-3p regulates the NSCLC immune microenvironment and suggested its potential as a novel therapeutic target and biomarker for immunotherapy.

We further explored the molecular basis of miR-4664-3p regulation of the tumor immune microenvironment and found that its target gene PRKCB was significantly downregulated in NSCLC, and was negatively correlated with miR-4664-3p expression. High PRKCB expression was associated with better survival outcomes, which is consistent with previous research findings (37, 38). PRKCB is a member of the protein kinase C (PRKC) family, consisting of several serine/threonine kinases, with its main function being the phosphorylation of proteins in various signal transduction pathways (39, 40). PRKCB is downregulated in multiple tumor tissues and is believed to play a unique role in cancer-related processes (41–44). Genetic alterations of PRKCB have been reported in NSCLC, suggesting its involvement in the progression of lung adenocarcinoma (45, 46). PRKCB is involved in cancer-related pathways and immune cell receptor signaling pathways, such as B cell receptor signaling, T cell receptor signaling, and VEGF signaling, and is associated with immune cell infiltration in NSCLC (36, 37). Our CIBERSORT-Absolute (CIBERSORT-ABS) analysis revealed that PRKCB expression was positively correlated with CD8^+^ T cell infiltration. Taken together, these results suggest that miR-4664-3p may promote immune evasion and tumor progression by suppressing PRKCB and weakening CD8^+^ T cell activity. Notably, PRKCB demonstrates high diagnostic efficacy in NSCLC (with an AUC as high as 0.937), indicating its promising clinical translational potential for NSCLC diagnosis. However, as a potential biomarker for NSCLC, miR-4664-3p may have off-target effects. Given its ability to regulate multiple target genes, these effects may be influenced by the molecular background and immune microenvironment, which could impact its specificity as a single biomarker. Nevertheless, based on the analysis from the TCGA database and *in vivo* and *in vitro* experiments, miR-4664-3p still shows potential in the diagnosis and prognosis assessment of NSCLC. Considering its multi-target characteristics, future studies could improve its specificity and clinical predictive efficacy by combining miR-4664-3p with other molecular markers or immune features, particularly in applications related to immunotherapy.

This study still has certain limitations. First, this study primarily relies on transcriptomic data from public databases, lacking validation in clinical cohorts of NSCLC (especially patients undergoing immunotherapy). Future research should combine large-scale clinical samples to further confirm the clinical relevance of miR-4664-3p as a therapeutic response marker. Second, issues such as the limited sample size, tissue heterogeneity, and uneven spatial distribution remain unaddressed, which may weaken the robustness and generalizability of the results. TCGA samples collected and processed by different centers may have quality differences, which could lead to bias in miRNA expression and immune-related features. To overcome these challenges, future research needs to integrate larger-scale, multi-center data and utilize emerging methods such as spatial transcriptomics and single-cell sequencing to more accurately characterize the role of miR-4664-3p in the tumor immune microenvironment.

## 5 Conclusions

In conclusion, through systematic analysis and *in vitro* and *in vivo* experimental validation, this study reveals the expression characteristics and clinical significance of miR-4664-3p in NSCLC. miR-4664-3p promotes tumor progression by inhibiting PRKCB, which affects the infiltration and activity of CD8^+^ T cells. This molecule has potential application value in the diagnosis, prognosis, and prediction of immunotherapy response in NSCLC and is expected to become an important target for future personalized treatment.

## Data Availability

The raw data supporting the conclusions of this article will be made available by the authors, without undue reservation.
