# Supplementary material for: MiR-4664-3p as a potential diagnostic, prognostic, and immunotherapeutic biomarker in NSCLC: modulation of tumor progression through CD8 + T cell regulation
Source: Front Oncol. 2025 Nov 4;15:1642999. doi: 10.3389/fonc.2025.1642999 (PMC12623161; doi:10.3389/fonc.2025.1642999)
Supplement: Supplementary file 1 [file DataSheet1.docx]

Supplementary Material

## Supplementary Table 1. Basic clinical characteristics of NSCLC patients

| Clinical variable | Group | Count |
| --- | --- | --- |
| Age | <=65 | 431 |
| Age | >65 | 567 |
|  | Unknown | 28 |
| Gender | FEMALE | 411 |
| Gender | MALE | 615 |
| Stage | Stage I | 524 |
| Stage | Stage II | 287 |
| Stage | Stage III | 170 |
| Stage | Stage IV | 33 |
|  | Unknown | 12 |
| T | T1 | 287 |
| T | T2 | 576 |
| T | T3 | 118 |
| T | T4 | 43 |
|  | Unknown | 3 |
| M | M0 | 767 |
| M | M1 | 32 |
|  | Unknown | 227 |
| N | N0 | 655 |
| N | N1 | 231 |
| N | N2 | 115 |
| N | N3 | 7 |
|  | Unknown | 18 |
| Survival status | Alive | 620 |
|  | Dead | 406 |
| Survival time | Median (IQR) days | 658(366,1182) |

**Supplementary Table 2. Primer sequences used for miRNA qRT-PCR**

| Sequence Name | Primer Sequence (5’-3’) |
| --- | --- |
| has-miR-4664-3p | Forward Primer GTCCTTCCGGTCTGTGAGCCCC |
|  | Reverse Primer GTGCAGGGTCCGAGGT |
| U6 | Forward Primer CGCTTCGGCAGCACATATAC |
|  | Reverse Primer TTCACGAATTTGCGTGTCATC |

**Supplementary Table 3.** **Primer sequences used for mRNA qRT-PCR**

| Gene | Primer Sequence (5’-3’) |
| --- | --- |
| β-actin | F:TTCCTTCCTGGGCATGGAGTC |
|  | R: TCTTCATTGTGCTGGGTGCC |
| PRKCB | F: CTTCAAGCAGCCCACCTTCT |
|  | R: ACAAAGCAGCAAACTTGGCA |

## Supplementary Table 4. Differentially expressed miRNAs in NSCLC tumor vs. normal tissues TCGA

| miRNA | conMean | treatMean | logFC | pValue | fdr |
| --- | --- | --- | --- | --- | --- |
| hsa-miR-1269b | 0.103343 | 41.20946 | 8.639394 | 4.76E-11 | 1.10E-10 |
| hsa-miR-105-5p | 0.863081 | 106.9386 | 6.953072 | 6.67E-22 | 3.34E-21 |
| hsa-miR-4652-5p | 0.048259 | 4.487341 | 6.538924 | 6.20E-31 | 5.99E-30 |
| hsa-miR-767-5p | 0.717293 | 55.43173 | 6.272006 | 1.47E-16 | 5.04E-16 |
| hsa-miR-196a-5p | 1.673962 | 128.8156 | 6.265896 | 7.67E-35 | 1.16E-33 |
| hsa-miR-1269a | 5.243377 | 325.879 | 5.957696 | 3.10E-30 | 2.79E-29 |
| hsa-miR-944 | 1.648706 | 94.66242 | 5.843385 | 1.11E-14 | 3.38E-14 |
| hsa-miR-31-5p | 0.532877 | 30.15504 | 5.822451 | 2.10E-33 | 2.51E-32 |
| hsa-miR-301b-5p | 0.034348 | 1.772888 | 5.689731 | 7.73E-34 | 9.96E-33 |
| hsa-miR-205-3p | 0.07699 | 3.871566 | 5.652095 | 2.22E-18 | 8.53E-18 |
| hsa-miR-205-5p | 162.2745 | 7956.877 | 5.615694 | 5.26E-20 | 2.30E-19 |
| hsa-miR-577 | 0.40849 | 17.48757 | 5.419886 | 2.75E-37 | 5.64E-36 |
| hsa-miR-4788 | 0.219001 | 8.123506 | 5.213094 | 2.52E-05 | 3.80E-05 |
| hsa-miR-9-5p | 156.3122 | 5395.569 | 5.109272 | 2.75E-47 | 1.64E-45 |
| hsa-miR-31-3p | 0.446473 | 15.10971 | 5.080758 | 8.10E-29 | 6.65E-28 |
| hsa-miR-196b-5p | 12.38059 | 358.2576 | 4.854845 | 6.84E-29 | 5.69E-28 |
| hsa-miR-9-3p | 0.304721 | 8.200062 | 4.750076 | 4.67E-33 | 5.20E-32 |
| hsa-miR-210-3p | 78.0667 | 2056.361 | 4.719243 | 2.65E-50 | 4.36E-48 |
| hsa-miR-3662 | 0.034015 | 0.878192 | 4.690284 | 3.85E-19 | 1.56E-18 |
| hsa-miR-7974 | 0.020121 | 0.458459 | 4.510046 | 3.47E-16 | 1.14E-15 |
| hsa-miR-891a-5p | 2.187054 | 49.3755 | 4.496735 | 7.84E-12 | 1.93E-11 |
| hsa-miR-6510-3p | 0.728575 | 14.55334 | 4.320129 | 2.18E-19 | 8.94E-19 |
| hsa-miR-137-3p | 0.158218 | 2.565822 | 4.019433 | 2.47E-17 | 9.05E-17 |
| hsa-miR-147b-3p | 0.132724 | 2.04431 | 3.945111 | 2.97E-29 | 2.50E-28 |
| hsa-miR-708-5p | 6.470056 | 95.63102 | 3.885628 | 1.24E-48 | 1.02E-46 |
| hsa-miR-301b-3p | 0.171417 | 2.408189 | 3.812366 | 5.75E-36 | 1.02E-34 |
| hsa-miR-183-3p | 0.18152 | 2.36712 | 3.70493 | 2.28E-41 | 6.81E-40 |
| hsa-miR-5579-3p | 0.0589 | 0.745055 | 3.661014 | 8.51E-12 | 2.09E-11 |
| hsa-miR-4766-3p | 0.056939 | 0.675317 | 3.568065 | 1.09E-18 | 4.31E-18 |
| hsa-miR-4664-5p | 0.028147 | 0.315737 | 3.487658 | 2.35E-13 | 6.43E-13 |
| hsa-miR-323b-3p | 0.911071 | 9.836427 | 3.432499 | 2.11E-29 | 1.80E-28 |
| hsa-miR-301a-5p | 0.907394 | 9.635863 | 3.408613 | 5.29E-39 | 1.29E-37 |
| hsa-miR-20b-3p | 0.249111 | 2.499214 | 3.326612 | 2.86E-13 | 7.79E-13 |
| hsa-miR-33b-5p | 0.662638 | 6.415923 | 3.275363 | 5.06E-38 | 1.15E-36 |
| hsa-miR-96-5p | 3.597074 | 34.42039 | 3.258367 | 7.11E-52 | 1.56E-49 |
| hsa-miR-487a-3p | 0.095738 | 0.911991 | 3.251858 | 2.25E-08 | 4.22E-08 |
| hsa-miR-708-3p | 22.24474 | 199.328 | 3.163608 | 2.94E-43 | 1.14E-41 |
| hsa-miR-6728-5p | 0.05194 | 0.454218 | 3.128464 | 1.63E-13 | 4.59E-13 |
| hsa-miR-675-3p | 10.4572 | 90.82504 | 3.118594 | 4.19E-09 | 8.24E-09 |
| hsa-miR-224-5p | 14.47891 | 125.0673 | 3.11068 | 3.43E-27 | 2.53E-26 |
| hsa-miR-183-5p | 1858.983 | 15260.56 | 3.037223 | 8.29E-53 | 5.45E-50 |
| hsa-miR-135b-5p | 8.110773 | 66.04178 | 3.025468 | 1.68E-36 | 3.06E-35 |
| hsa-miR-1248 | 0.134655 | 1.085023 | 3.010382 | 8.17E-17 | 2.82E-16 |
| hsa-miR-545-5p | 0.060917 | 0.489565 | 3.006587 | 3.71E-17 | 1.34E-16 |
| hsa-miR-1537-5p | 0.038479 | 0.306399 | 2.993263 | 7.75E-12 | 1.91E-11 |
| hsa-miR-616-5p | 0.536012 | 4.03625 | 2.912678 | 1.07E-32 | 1.15E-31 |
| hsa-miR-215-5p | 3.827453 | 28.72697 | 2.907949 | 0.006434 | 0.007991 |
| hsa-miR-556-5p | 0.0555 | 0.412785 | 2.894818 | 5.78E-10 | 1.23E-09 |
| hsa-miR-556-3p | 0.073359 | 0.543141 | 2.888286 | 3.13E-16 | 1.03E-15 |
| hsa-miR-135b-3p | 0.211461 | 1.541835 | 2.866184 | 5.00E-24 | 2.83E-23 |
| hsa-miR-323a-3p | 1.101104 | 7.924823 | 2.847427 | 1.18E-18 | 4.66E-18 |
| hsa-miR-155-3p | 0.117654 | 0.836398 | 2.829638 | 4.31E-21 | 2.04E-20 |
| hsa-miR-33a-5p | 6.875457 | 46.54301 | 2.759037 | 1.61E-34 | 2.26E-33 |
| hsa-miR-431-5p | 0.649507 | 4.323239 | 2.734695 | 4.96E-10 | 1.07E-09 |
| hsa-miR-539-5p | 1.202421 | 7.92269 | 2.720048 | 2.04E-24 | 1.19E-23 |
| hsa-miR-4454 | 0.070342 | 0.460637 | 2.711166 | 3.32E-08 | 6.12E-08 |
| hsa-miR-153-5p | 2.816935 | 18.13349 | 2.686459 | 9.92E-32 | 1.00E-30 |
| hsa-miR-7112-3p | 0.061031 | 0.382305 | 2.647118 | 8.28E-11 | 1.87E-10 |
| hsa-miR-4664-3p | 0.099355 | 0.612027 | 2.622931 | 5.01E-17 | 1.76E-16 |
| hsa-miR-489-3p | 0.155411 | 0.949792 | 2.611527 | 0.002591 | 0.003358 |
| hsa-miR-503-5p | 1.594843 | 9.655638 | 2.597957 | 2.74E-38 | 6.44E-37 |
| hsa-miR-26a-1-3p | 0.464481 | 2.804161 | 2.593878 | 1.91E-14 | 5.66E-14 |
| hsa-miR-579-5p | 0.179741 | 1.081529 | 2.589077 | 1.55E-21 | 7.59E-21 |
| hsa-miR-192-5p | 284.9415 | 1712.751 | 2.587577 | 3.36E-18 | 1.27E-17 |
| hsa-miR-130b-3p | 4.811468 | 28.11563 | 2.546823 | 1.97E-49 | 1.91E-47 |
| hsa-miR-409-5p | 2.066709 | 12.01677 | 2.539642 | 6.59E-25 | 3.90E-24 |
| hsa-miR-129-5p | 3.905198 | 22.64129 | 2.535488 | 0.000325 | 0.000453 |
| hsa-miR-182-5p | 4809.413 | 27732.5 | 2.527645 | 9.42E-50 | 1.24E-47 |
| hsa-miR-744-3p | 4.31749 | 24.7245 | 2.517676 | 1.17E-35 | 1.98E-34 |
| hsa-miR-570-3p | 0.166823 | 0.949137 | 2.508295 | 1.41E-14 | 4.21E-14 |
| hsa-miR-615-3p | 0.658646 | 3.68893 | 2.485628 | 2.04E-18 | 7.92E-18 |
| hsa-miR-3664-5p | 0.078525 | 0.439154 | 2.483507 | 3.69E-08 | 6.80E-08 |
| hsa-miR-1277-3p | 0.313079 | 1.74699 | 2.480273 | 5.25E-26 | 3.42E-25 |
| hsa-miR-4668-3p | 0.577254 | 3.209279 | 2.47497 | 1.20E-34 | 1.72E-33 |
| hsa-miR-548v | 0.443454 | 2.422935 | 2.449899 | 4.91E-26 | 3.22E-25 |
| hsa-miR-20a-3p | 1.694615 | 9.141599 | 2.431489 | 1.21E-37 | 2.56E-36 |
| hsa-miR-671-5p | 1.200726 | 6.370088 | 2.407407 | 8.37E-43 | 2.75E-41 |
| hsa-miR-203a-5p | 0.354307 | 1.827589 | 2.366871 | 5.89E-08 | 1.07E-07 |
| hsa-miR-141-3p | 265.7443 | 1338.145 | 2.332124 | 3.94E-45 | 2.16E-43 |
| hsa-miR-381-5p | 0.132175 | 0.659916 | 2.319833 | 3.47E-09 | 6.91E-09 |
| hsa-miR-3176 | 0.070212 | 0.344496 | 2.294689 | 6.78E-10 | 1.42E-09 |
| hsa-miR-548au-3p | 0.09434 | 0.462338 | 2.293001 | 2.37E-11 | 5.62E-11 |
| hsa-miR-149-5p | 29.74485 | 142.6855 | 2.262127 | 1.36E-16 | 4.67E-16 |
| hsa-miR-130b-5p | 5.255588 | 25.0849 | 2.254895 | 4.40E-40 | 1.16E-38 |
| hsa-miR-3136-5p | 0.161919 | 0.769767 | 2.24915 | 1.18E-19 | 4.96E-19 |
| hsa-miR-628-5p | 5.665668 | 26.84745 | 2.244467 | 5.56E-20 | 2.42E-19 |
| hsa-miR-136-5p | 6.513908 | 30.78864 | 2.240803 | 1.17E-26 | 8.23E-26 |
| hsa-miR-6815-3p | 0.061335 | 0.27927 | 2.186873 | 2.68E-07 | 4.66E-07 |
| hsa-miR-5571-3p | 0.074447 | 0.33871 | 2.185772 | 8.41E-09 | 1.64E-08 |
| hsa-miR-376a-3p | 0.349982 | 1.585939 | 2.179985 | 0.000701 | 0.000951 |
| hsa-miR-1307-5p | 118.9935 | 530.0798 | 2.155326 | 1.43E-30 | 1.34E-29 |
| hsa-miR-345-5p | 6.727144 | 29.87097 | 2.150678 | 7.74E-35 | 1.16E-33 |
| hsa-miR-103a-2-5p | 0.54276 | 2.408959 | 2.150023 | 7.82E-23 | 4.25E-22 |
| hsa-miR-203a-3p | 5268.054 | 23229.03 | 2.140587 | 3.12E-07 | 5.39E-07 |
| hsa-miR-365a-5p | 0.205595 | 0.905917 | 2.139574 | 1.31E-19 | 5.44E-19 |
| hsa-miR-200a-5p | 163.8021 | 715.7463 | 2.127495 | 9.37E-43 | 2.93E-41 |
| hsa-miR-452-3p | 2.317297 | 9.983238 | 2.107065 | 3.39E-14 | 9.81E-14 |
| hsa-miR-301a-3p | 3.66833 | 15.78482 | 2.105342 | 2.02E-32 | 2.14E-31 |
| hsa-miR-200a-3p | 90.28247 | 387.7426 | 2.102581 | 3.81E-37 | 7.59E-36 |
| hsa-miR-376c-5p | 0.1585 | 0.680571 | 2.102264 | 1.20E-09 | 2.48E-09 |
| hsa-miR-541-3p | 0.26169 | 1.119111 | 2.096421 | 0.003032 | 0.003898 |
| hsa-miR-629-3p | 2.33182 | 9.944493 | 2.092441 | 7.80E-36 | 1.35E-34 |
| hsa-miR-4491 | 0.341403 | 1.425572 | 2.061991 | 3.64E-11 | 8.51E-11 |
| hsa-miR-127-5p | 57.11111 | 236.8243 | 2.051974 | 1.23E-25 | 7.76E-25 |
| hsa-miR-376b-5p | 0.156984 | 0.648572 | 2.046654 | 3.62E-09 | 7.15E-09 |
| hsa-miR-1910-5p | 0.175137 | 0.720362 | 2.040235 | 1.33E-12 | 3.44E-12 |
| hsa-miR-187-3p | 10.90913 | 44.57601 | 2.030732 | 5.84E-07 | 9.89E-07 |
| hsa-miR-141-5p | 200.774 | 819.3074 | 2.028832 | 7.93E-43 | 2.74E-41 |
| hsa-miR-3170 | 0.365538 | 1.486415 | 2.023746 | 6.60E-23 | 3.64E-22 |
| hsa-miR-494-3p | 0.375669 | 1.527028 | 2.023192 | 9.26E-09 | 1.79E-08 |
| hsa-miR-412-5p | 1.478526 | 5.968617 | 2.013237 | 6.32E-07 | 1.06E-06 |
| hsa-miR-7-5p | 0.844089 | 3.393006 | 2.007097 | 2.16E-15 | 6.71E-15 |
| hsa-miR-581 | 0.146738 | 0.577771 | 1.977255 | 3.06E-12 | 7.78E-12 |
| hsa-miR-203b-3p | 32.03099 | 125.652 | 1.971893 | 1.30E-11 | 3.15E-11 |
| hsa-miR-377-3p | 0.36253 | 1.406631 | 1.956073 | 8.22E-11 | 1.87E-10 |
| hsa-miR-376a-5p | 0.306117 | 1.185013 | 1.952749 | 2.23E-07 | 3.89E-07 |
| hsa-miR-376b-3p | 0.39377 | 1.520393 | 1.949019 | 1.55E-07 | 2.75E-07 |
| hsa-miR-1287-3p | 2.468512 | 9.529078 | 1.948695 | 2.25E-28 | 1.74E-27 |
| hsa-miR-154-3p | 0.880337 | 3.393714 | 1.946737 | 1.25E-08 | 2.42E-08 |
| hsa-miR-424-5p | 26.1772 | 100.5531 | 1.941575 | 2.89E-35 | 4.52E-34 |
| hsa-miR-345-3p | 0.173303 | 0.665657 | 1.941479 | 7.93E-11 | 1.81E-10 |
| hsa-miR-2277-5p | 0.383257 | 1.452231 | 1.921885 | 1.71E-22 | 8.98E-22 |
| hsa-miR-188-3p | 0.417803 | 1.582949 | 1.921719 | 2.29E-19 | 9.35E-19 |
| hsa-miR-5581-3p | 0.08033 | 0.303094 | 1.915746 | 1.71E-07 | 3.01E-07 |
| hsa-miR-429 | 42.59443 | 158.8803 | 1.899203 | 4.03E-33 | 4.57E-32 |
| hsa-miR-551b-3p | 2.952733 | 10.99816 | 1.89714 | 0.00141 | 0.001867 |
| hsa-miR-627-5p | 0.536611 | 1.952072 | 1.863057 | 2.15E-22 | 1.10E-21 |
| hsa-miR-7-1-3p | 6.232636 | 22.53008 | 1.853938 | 9.92E-41 | 2.83E-39 |
| hsa-miR-219a-5p | 0.423821 | 1.528208 | 1.850316 | 5.05E-16 | 1.63E-15 |
| hsa-miR-296-3p | 0.605723 | 2.160672 | 1.834749 | 7.03E-12 | 1.75E-11 |
| hsa-miR-548d-3p | 0.087913 | 0.308946 | 1.813204 | 3.29E-05 | 4.92E-05 |
| hsa-miR-431-3p | 5.281736 | 18.50358 | 1.808721 | 0.000261 | 0.000367 |
| hsa-miR-590-5p | 5.973048 | 20.91831 | 1.808227 | 5.83E-32 | 5.98E-31 |
| hsa-miR-561-5p | 0.456642 | 1.595484 | 1.804858 | 5.10E-05 | 7.57E-05 |
| hsa-miR-589-3p | 1.125978 | 3.929197 | 1.803056 | 2.76E-25 | 1.71E-24 |
| hsa-miR-616-3p | 0.46837 | 1.623238 | 1.793153 | 1.52E-26 | 1.05E-25 |
| hsa-miR-19a-3p | 11.09244 | 38.24624 | 1.785741 | 2.03E-22 | 1.05E-21 |
| hsa-miR-106a-5p | 7.426646 | 25.60218 | 1.785484 | 2.89E-14 | 8.41E-14 |
| hsa-miR-369-3p | 2.794387 | 9.537829 | 1.771129 | 6.40E-10 | 1.35E-09 |
| hsa-let-7g-3p | 13.45403 | 45.33609 | 1.752621 | 2.07E-32 | 2.16E-31 |
| hsa-miR-181b-2-3p | 1.014234 | 3.400885 | 1.74552 | 4.54E-22 | 2.29E-21 |
| hsa-miR-889-3p | 7.067811 | 23.69859 | 1.745466 | 2.01E-08 | 3.79E-08 |
| hsa-miR-653-5p | 8.521007 | 28.4148 | 1.737547 | 0.007934 | 0.009707 |
| hsa-miR-450a-5p | 2.968585 | 9.832704 | 1.727813 | 2.27E-27 | 1.73E-26 |
| hsa-miR-548s | 0.139386 | 0.460344 | 1.723626 | 4.83E-08 | 8.84E-08 |
| hsa-miR-142-3p | 1173.696 | 3873.174 | 1.722457 | 6.45E-25 | 3.85E-24 |
| hsa-miR-627-3p | 0.078621 | 0.259359 | 1.721963 | 4.35E-05 | 6.49E-05 |
| hsa-miR-3140-3p | 0.077886 | 0.254795 | 1.709893 | 5.43E-06 | 8.58E-06 |
| hsa-miR-3677-3p | 1.877325 | 6.115751 | 1.703851 | 7.93E-19 | 3.16E-18 |
| hsa-miR-450b-5p | 5.447927 | 17.63154 | 1.694379 | 3.52E-26 | 2.36E-25 |
| hsa-miR-93-3p | 4.91636 | 15.79491 | 1.683797 | 3.63E-31 | 3.56E-30 |
| hsa-miR-639 | 0.22164 | 0.707868 | 1.675264 | 2.09E-14 | 6.17E-14 |
| hsa-miR-493-3p | 2.357427 | 7.49592 | 1.668892 | 2.78E-10 | 6.13E-10 |
| hsa-miR-200c-5p | 13.9106 | 44.01171 | 1.661703 | 3.50E-33 | 4.11E-32 |
| hsa-miR-193b-3p | 32.26802 | 101.8991 | 1.658965 | 2.74E-27 | 2.07E-26 |
| hsa-miR-186-3p | 0.104891 | 0.330329 | 1.655013 | 8.13E-07 | 1.35E-06 |
| hsa-miR-153-3p | 0.550894 | 1.732759 | 1.653224 | 2.94E-11 | 6.95E-11 |
| hsa-miR-491-3p | 0.302038 | 0.929455 | 1.621655 | 5.43E-13 | 1.46E-12 |
| hsa-miR-625-5p | 1.4793 | 4.526124 | 1.613361 | 9.50E-27 | 6.78E-26 |
| hsa-miR-148b-5p | 0.92365 | 2.820852 | 1.610714 | 8.31E-30 | 7.19E-29 |
| hsa-miR-409-3p | 7.914971 | 24.14675 | 1.609173 | 3.70E-16 | 1.21E-15 |
| hsa-miR-3691-5p | 0.188662 | 0.563145 | 1.577701 | 4.72E-07 | 8.11E-07 |
| hsa-miR-496 | 0.848348 | 2.530571 | 1.576735 | 1.39E-06 | 2.29E-06 |
| hsa-miR-651-5p | 1.988196 | 5.921016 | 1.574385 | 1.11E-14 | 3.38E-14 |
| hsa-miR-937-3p | 2.020739 | 6.011065 | 1.572737 | 1.47E-19 | 6.05E-19 |
| hsa-miR-4797-3p | 0.267599 | 0.794829 | 1.570573 | 7.38E-06 | 1.15E-05 |
| hsa-miR-3200-3p | 2.395554 | 7.103372 | 1.568145 | 7.08E-18 | 2.64E-17 |
| hsa-miR-134-5p | 128.5848 | 380.9082 | 1.566724 | 2.17E-08 | 4.08E-08 |
| hsa-miR-758-3p | 5.653698 | 16.40713 | 1.537056 | 1.56E-07 | 2.76E-07 |
| hsa-miR-21-5p | 117958 | 340614.6 | 1.529867 | 7.14E-45 | 3.35E-43 |
| hsa-miR-21-3p | 1458.228 | 4181.127 | 1.519676 | 4.97E-34 | 6.53E-33 |
| hsa-miR-455-3p | 102.402 | 293.127 | 1.517281 | 1.01E-28 | 8.07E-28 |
| hsa-miR-3129-3p | 0.120154 | 0.343156 | 1.513976 | 9.95E-06 | 1.55E-05 |
| hsa-miR-592 | 1.335745 | 3.81323 | 1.513369 | 3.35E-12 | 8.47E-12 |
| hsa-miR-17-5p | 206.668 | 589.2052 | 1.511455 | 5.17E-25 | 3.15E-24 |
| hsa-miR-130a-5p | 0.482651 | 1.370735 | 1.505898 | 7.04E-13 | 1.86E-12 |
| hsa-miR-4473 | 0.251937 | 0.705582 | 1.485751 | 3.06E-09 | 6.11E-09 |
| hsa-miR-4638-3p | 0.373186 | 1.044754 | 1.485198 | 2.88E-20 | 1.32E-19 |
| hsa-miR-4443 | 0.285349 | 0.78624 | 1.462241 | 6.73E-08 | 1.22E-07 |
| hsa-miR-656-3p | 0.249737 | 0.686304 | 1.458438 | 0.000301 | 0.000422 |
| hsa-miR-4791 | 0.26497 | 0.72814 | 1.458387 | 7.58E-09 | 1.48E-08 |
| hsa-miR-3684 | 0.177185 | 0.485793 | 1.455081 | 5.03E-07 | 8.64E-07 |
| hsa-miR-411-3p | 0.572871 | 1.568179 | 1.452808 | 0.018896 | 0.02245 |
| hsa-miR-1291 | 0.258262 | 0.706507 | 1.451869 | 1.69E-09 | 3.49E-09 |
| hsa-miR-4661-5p | 1.983403 | 5.402599 | 1.445676 | 3.85E-18 | 1.45E-17 |
| hsa-miR-493-5p | 4.017217 | 10.93742 | 1.445005 | 8.28E-11 | 1.87E-10 |
| hsa-miR-148a-3p | 21498.25 | 57982.2 | 1.431391 | 2.09E-28 | 1.64E-27 |
| hsa-miR-590-3p | 2.777187 | 7.346685 | 1.403469 | 1.74E-25 | 1.09E-24 |
| hsa-miR-543 | 0.464326 | 1.223993 | 1.398384 | 0.033155 | 0.038622 |
| hsa-miR-487b-3p | 2.387642 | 6.285182 | 1.396368 | 5.13E-06 | 8.14E-06 |
| hsa-miR-539-3p | 0.297896 | 0.778088 | 1.385124 | 0.037579 | 0.043698 |
| hsa-miR-299-3p | 0.266374 | 0.695234 | 1.384044 | 0.032893 | 0.038385 |
| hsa-miR-760 | 1.18615 | 3.091522 | 1.382031 | 5.00E-17 | 1.76E-16 |
| hsa-miR-98-3p | 0.59687 | 1.539872 | 1.36732 | 4.13E-16 | 1.34E-15 |
| hsa-miR-1284 | 0.125248 | 0.322566 | 1.364809 | 8.73E-05 | 0.000127 |
| hsa-miR-154-5p | 1.779874 | 4.57383 | 1.361628 | 2.56E-09 | 5.18E-09 |
| hsa-miR-4724-5p | 0.370398 | 0.951158 | 1.360607 | 0.000205 | 0.000293 |
| hsa-miR-3677-5p | 0.613412 | 1.572326 | 1.357973 | 3.55E-13 | 9.61E-13 |
| hsa-miR-93-5p | 2637.592 | 6744.351 | 1.354458 | 2.91E-20 | 1.32E-19 |
| hsa-miR-224-3p | 2.05769 | 5.260032 | 1.354046 | 8.63E-09 | 1.67E-08 |
| hsa-miR-4746-5p | 1.547316 | 3.95512 | 1.353954 | 7.98E-16 | 2.53E-15 |
| hsa-miR-942-3p | 0.327254 | 0.836497 | 1.35395 | 1.14E-06 | 1.88E-06 |
| hsa-miR-219b-3p | 0.185526 | 0.47335 | 1.351285 | 7.60E-07 | 1.27E-06 |
| hsa-miR-200b-3p | 321.3459 | 814.2261 | 1.341302 | 1.30E-26 | 9.10E-26 |
| hsa-miR-454-3p | 3.408321 | 8.634681 | 1.341082 | 3.24E-27 | 2.42E-26 |
| hsa-miR-6783-3p | 0.150962 | 0.382206 | 1.340167 | 9.52E-06 | 1.48E-05 |
| hsa-miR-20b-5p | 13.63836 | 34.48983 | 1.338501 | 0.003472 | 0.004429 |
| hsa-miR-143-5p | 32.37373 | 81.64878 | 1.334608 | 3.06E-17 | 1.11E-16 |
| hsa-miR-3609 | 0.201688 | 0.50824 | 1.333387 | 1.14E-05 | 1.76E-05 |
| hsa-miR-337-3p | 11.6284 | 29.04616 | 1.320695 | 4.92E-11 | 1.14E-10 |
| hsa-miR-151a-5p | 46.3292 | 115.5286 | 1.318256 | 5.05E-37 | 9.48E-36 |
| hsa-miR-628-3p | 0.746844 | 1.855368 | 1.312826 | 3.20E-11 | 7.55E-11 |
| hsa-miR-655-3p | 0.99938 | 2.482343 | 1.312597 | 0.000718 | 0.000972 |
| hsa-miR-382-5p | 7.661687 | 19.01036 | 1.311051 | 4.98E-10 | 1.07E-09 |
| hsa-miR-495-3p | 3.54199 | 8.700992 | 1.29662 | 0.00413 | 0.005248 |
| hsa-miR-29b-1-5p | 2.707555 | 6.640872 | 1.294382 | 2.19E-21 | 1.06E-20 |
| hsa-miR-5706 | 0.145997 | 0.357444 | 1.29178 | 1.42E-05 | 2.18E-05 |
| hsa-miR-188-5p | 1.052183 | 2.575237 | 1.291319 | 2.19E-13 | 6.06E-13 |
| hsa-miR-550a-5p | 2.225629 | 5.433733 | 1.287731 | 2.08E-18 | 8.04E-18 |
| hsa-miR-18a-3p | 0.974476 | 2.379043 | 1.287683 | 2.48E-17 | 9.06E-17 |
| hsa-miR-425-5p | 99.11087 | 238.3123 | 1.265739 | 1.04E-24 | 6.10E-24 |
| hsa-miR-370-3p | 3.524463 | 8.430347 | 1.258188 | 7.59E-08 | 1.37E-07 |
| hsa-miR-432-5p | 4.882185 | 11.62249 | 1.251321 | 0.016469 | 0.019638 |
| hsa-miR-5699-3p | 0.171863 | 0.407637 | 1.246029 | 1.28E-09 | 2.65E-09 |
| hsa-miR-382-3p | 0.949913 | 2.216249 | 1.222253 | 5.84E-05 | 8.63E-05 |
| hsa-miR-643 | 0.213074 | 0.495693 | 1.21809 | 2.64E-06 | 4.26E-06 |
| hsa-miR-29a-5p | 4.333409 | 9.942691 | 1.198134 | 3.46E-21 | 1.66E-20 |
| hsa-miR-1301-3p | 7.971404 | 18.22156 | 1.192741 | 1.02E-31 | 1.01E-30 |
| hsa-miR-625-3p | 140.7928 | 319.8469 | 1.183808 | 5.69E-27 | 4.16E-26 |
| hsa-miR-576-5p | 6.080671 | 13.708 | 1.172716 | 8.09E-23 | 4.36E-22 |
| hsa-miR-18a-5p | 9.613778 | 21.64264 | 1.170701 | 1.00E-10 | 2.24E-10 |
| hsa-miR-629-5p | 71.36788 | 160.5722 | 1.169875 | 1.13E-25 | 7.20E-25 |
| hsa-miR-324-5p | 15.58528 | 35.05498 | 1.169435 | 1.26E-23 | 7.02E-23 |
| hsa-let-7a-2-3p | 5.823449 | 13.04086 | 1.163094 | 1.10E-12 | 2.88E-12 |
| hsa-miR-4444 | 0.534368 | 1.196007 | 1.16232 | 1.14E-12 | 2.95E-12 |
| hsa-miR-769-3p | 1.162309 | 2.586366 | 1.153933 | 1.20E-19 | 5.04E-19 |
| hsa-miR-7705 | 0.598446 | 1.330436 | 1.152606 | 4.15E-09 | 8.18E-09 |
| hsa-miR-939-5p | 0.573831 | 1.271999 | 1.148399 | 7.62E-15 | 2.33E-14 |
| hsa-miR-27b-5p | 11.37342 | 24.96143 | 1.134034 | 6.84E-26 | 4.40E-25 |
| hsa-miR-6516-3p | 0.120294 | 0.262316 | 1.12474 | 2.93E-05 | 4.39E-05 |
| hsa-miR-503-3p | 0.531209 | 1.155308 | 1.120926 | 6.29E-19 | 2.52E-18 |
| hsa-miR-4449 | 0.472326 | 1.022362 | 1.114053 | 2.01E-14 | 5.95E-14 |
| hsa-miR-3944-3p | 0.154139 | 0.331631 | 1.105345 | 5.47E-07 | 9.31E-07 |
| hsa-miR-19b-1-5p | 4.633457 | 9.929442 | 1.099623 | 1.95E-16 | 6.50E-16 |
| hsa-miR-134-3p | 0.514341 | 1.098646 | 1.09493 | 8.10E-05 | 0.000119 |
| hsa-miR-5001-5p | 0.273729 | 0.581775 | 1.087713 | 2.41E-09 | 4.88E-09 |
| hsa-miR-210-5p | 0.753049 | 1.600346 | 1.087568 | 1.02E-19 | 4.34E-19 |
| hsa-let-7c-3p | 6.01214 | 12.72508 | 1.081724 | 8.67E-05 | 0.000127 |
| hsa-miR-152-5p | 0.348094 | 0.732369 | 1.073093 | 6.01E-05 | 8.86E-05 |
| hsa-miR-376c-3p | 3.019156 | 6.317905 | 1.065301 | 0.014251 | 0.017117 |
| hsa-miR-4326 | 3.051312 | 6.375935 | 1.063207 | 4.52E-10 | 9.83E-10 |
| hsa-miR-2355-3p | 3.239744 | 6.721947 | 1.052999 | 1.23E-13 | 3.50E-13 |
| hsa-miR-5001-3p | 0.196584 | 0.407515 | 1.051709 | 2.77E-05 | 4.17E-05 |
| hsa-miR-654-3p | 15.23449 | 31.41222 | 1.043985 | 0.01246 | 0.015048 |
| hsa-miR-3942-5p | 0.201278 | 0.414669 | 1.042769 | 0.022932 | 0.026952 |
| hsa-miR-24-2-5p | 15.74841 | 32.43687 | 1.042429 | 1.56E-17 | 5.77E-17 |
| hsa-miR-4762-3p | 0.116821 | 0.240274 | 1.040376 | 0.005308 | 0.006655 |
| hsa-miR-19b-3p | 73.72508 | 150.7813 | 1.03223 | 3.20E-15 | 9.89E-15 |
| hsa-miR-20a-5p | 183.3337 | 374.6042 | 1.030895 | 5.55E-10 | 1.18E-09 |
| hsa-miR-3127-3p | 0.133464 | 0.271879 | 1.026518 | 1.46E-06 | 2.39E-06 |
| hsa-miR-199b-3p | 1415.436 | 2881.591 | 1.025619 | 4.51E-17 | 1.61E-16 |
| hsa-miR-29b-3p | 446.6775 | 909.0946 | 1.025197 | 1.06E-17 | 3.95E-17 |
| hsa-miR-199a-3p | 1420.95 | 2888.237 | 1.023334 | 4.80E-17 | 1.70E-16 |
| hsa-miR-4677-3p | 5.207487 | 10.56263 | 1.02031 | 5.64E-21 | 2.65E-20 |
| hsa-miR-1229-3p | 0.426354 | 0.861606 | 1.014978 | 3.68E-10 | 8.08E-10 |
| hsa-miR-580-3p | 0.366624 | 0.739798 | 1.01283 | 1.23E-05 | 1.90E-05 |
| hsa-miR-452-5p | 74.7766 | 150.7805 | 1.011792 | 0.001745 | 0.002293 |
| hsa-miR-4645-3p | 0.197175 | 0.396417 | 1.007539 | 0.000204 | 0.000292 |
| hsa-miR-3187-3p | 0.18834 | 0.37803 | 1.005163 | 2.73E-05 | 4.11E-05 |
| hsa-miR-585-5p | 0.267322 | 0.133292 | -1.00398 | 6.68E-10 | 1.41E-09 |
| hsa-let-7b-5p | 32288.47 | 15923.07 | -1.0199 | 6.51E-20 | 2.78E-19 |
| hsa-miR-338-3p | 1511.659 | 708.0215 | -1.09427 | 2.38E-16 | 7.89E-16 |
| hsa-miR-27a-5p | 44.37591 | 20.4544 | -1.11737 | 7.79E-11 | 1.78E-10 |
| hsa-let-7a-5p | 59484.96 | 27369.22 | -1.11997 | 1.06E-34 | 1.54E-33 |
| hsa-let-7f-5p | 18772.49 | 8585.171 | -1.1287 | 8.85E-13 | 2.32E-12 |
| hsa-miR-6502-5p | 1.067159 | 0.48459 | -1.13894 | 1.80E-16 | 6.05E-16 |
| hsa-miR-125a-5p | 1205.564 | 543.2672 | -1.14997 | 4.91E-05 | 7.30E-05 |
| hsa-miR-145-3p | 73.13637 | 32.8123 | -1.15635 | 9.68E-34 | 1.22E-32 |
| hsa-miR-126-5p | 571.7283 | 255.6966 | -1.1609 | 2.43E-26 | 1.65E-25 |
| hsa-miR-6514-3p | 0.36934 | 0.163559 | -1.17513 | 0.002341 | 0.003052 |
| hsa-miR-211-5p | 0.67936 | 0.299148 | -1.18332 | 1.95E-09 | 4.01E-09 |
| hsa-miR-145-5p | 3256.3 | 1431.062 | -1.18615 | 3.54E-09 | 7.03E-09 |
| hsa-miR-5193 | 0.324626 | 0.142603 | -1.18677 | 0.000542 | 0.00074 |
| hsa-let-7c-5p | 3298.755 | 1444.281 | -1.19157 | 3.85E-34 | 5.27E-33 |
| hsa-miR-34c-3p | 86.08735 | 37.0148 | -1.2177 | 8.08E-09 | 1.58E-08 |
| hsa-let-7b-3p | 57.896 | 24.57824 | -1.23608 | 8.76E-12 | 2.14E-11 |
| hsa-miR-30d-5p | 23923.41 | 10096.28 | -1.2446 | 4.08E-34 | 5.48E-33 |
| hsa-miR-146b-3p | 462.3186 | 189.1848 | -1.28909 | 1.87E-13 | 5.21E-13 |
| hsa-miR-1294 | 0.65052 | 0.26364 | -1.30303 | 4.03E-14 | 1.16E-13 |
| hsa-miR-1247-3p | 49.08311 | 19.84683 | -1.30632 | 5.46E-24 | 3.07E-23 |
| hsa-miR-195-3p | 8.351155 | 3.316819 | -1.33218 | 4.47E-22 | 2.28E-21 |
| hsa-miR-101-3p | 29026.85 | 11363.15 | -1.35303 | 1.16E-30 | 1.10E-29 |
| hsa-miR-6507-5p | 0.415519 | 0.16233 | -1.35598 | 1.58E-16 | 5.35E-16 |
| hsa-miR-140-3p | 2276.301 | 875.8938 | -1.37786 | 6.67E-45 | 3.35E-43 |
| hsa-miR-1258 | 1.927589 | 0.739078 | -1.383 | 5.71E-25 | 3.44E-24 |
| hsa-miR-4777-3p | 1.322194 | 0.50208 | -1.39694 | 5.65E-19 | 2.28E-18 |
| hsa-miR-485-5p | 0.905496 | 0.34212 | -1.40421 | 0.000542 | 0.00074 |
| hsa-miR-190a-5p | 7.607476 | 2.848677 | -1.41713 | 1.50E-22 | 7.93E-22 |
| hsa-miR-4529-3p | 0.610279 | 0.228048 | -1.42013 | 2.43E-20 | 1.13E-19 |
| hsa-miR-607 | 0.280307 | 0.099701 | -1.49132 | 6.65E-13 | 1.77E-12 |
| hsa-miR-2110 | 4.656798 | 1.654827 | -1.49266 | 0.005108 | 0.006416 |
| hsa-miR-1247-5p | 19.66304 | 6.917214 | -1.50722 | 4.35E-16 | 1.41E-15 |
| hsa-miR-218-5p | 168.8418 | 59.38583 | -1.50748 | 8.89E-33 | 9.74E-32 |
| hsa-miR-4683 | 0.591401 | 0.20747 | -1.51123 | 1.25E-12 | 3.23E-12 |
| hsa-miR-195-5p | 97.10822 | 32.62429 | -1.57365 | 3.57E-33 | 4.11E-32 |
| hsa-miR-887-5p | 1.236991 | 0.412137 | -1.58564 | 5.86E-10 | 1.24E-09 |
| hsa-miR-1976 | 42.16497 | 12.99445 | -1.69815 | 5.22E-15 | 1.60E-14 |
| hsa-miR-3622a-3p | 0.661525 | 0.20078 | -1.72018 | 3.46E-06 | 5.54E-06 |
| hsa-miR-338-5p | 30.62324 | 9.129625 | -1.746 | 2.18E-40 | 5.97E-39 |
| hsa-miR-584-3p | 0.687126 | 0.196915 | -1.803 | 1.10E-20 | 5.13E-20 |
| hsa-miR-143-3p | 247385.2 | 70672.22 | -1.80754 | 2.00E-30 | 1.85E-29 |
| hsa-miR-1-3p | 36.46458 | 10.00769 | -1.86539 | 5.82E-30 | 5.10E-29 |
| hsa-miR-218-1-3p | 3.835653 | 1.037624 | -1.88619 | 1.20E-37 | 2.56E-36 |
| hsa-miR-4521 | 1.881517 | 0.496642 | -1.92162 | 1.37E-07 | 2.44E-07 |
| hsa-miR-326 | 57.14975 | 14.89336 | -1.94008 | 8.95E-12 | 2.18E-11 |
| hsa-miR-206 | 4.086776 | 1.008111 | -2.01931 | 1.82E-26 | 1.24E-25 |
| hsa-miR-504-5p | 5.341639 | 1.315876 | -2.02126 | 1.23E-05 | 1.90E-05 |
| hsa-miR-30a-5p | 63749.65 | 15543.05 | -2.03615 | 4.62E-37 | 8.92E-36 |
| hsa-miR-486-3p | 2.786586 | 0.62355 | -2.15992 | 7.95E-07 | 1.33E-06 |
| hsa-miR-139-5p | 162.8265 | 35.99112 | -2.17762 | 1.55E-35 | 2.55E-34 |
| hsa-miR-150-3p | 12.77242 | 2.804826 | -2.18705 | 6.15E-11 | 1.42E-10 |
| hsa-miR-6892-5p | 11.26014 | 2.468052 | -2.18978 | 8.49E-29 | 6.88E-28 |
| hsa-miR-605-5p | 1.742591 | 0.366063 | -2.25107 | 9.62E-08 | 1.73E-07 |
| hsa-miR-133a-3p | 31.95299 | 6.509651 | -2.2953 | 4.14E-39 | 1.05E-37 |
| hsa-miR-30a-3p | 20069.37 | 4064.406 | -2.30388 | 2.04E-49 | 1.91E-47 |
| hsa-miR-133b | 5.123025 | 0.961366 | -2.41384 | 2.47E-44 | 1.08E-42 |
| hsa-miR-144-3p | 64.04435 | 11.0921 | -2.52954 | 5.69E-30 | 5.05E-29 |
| hsa-miR-30c-2-3p | 172.9166 | 29.57053 | -2.54784 | 6.89E-52 | 1.56E-49 |
| hsa-miR-490-3p | 3.095326 | 0.499079 | -2.63275 | 7.88E-44 | 3.23E-42 |
| hsa-miR-451a | 4097.619 | 647.5101 | -2.66181 | 1.95E-33 | 2.37E-32 |
| hsa-miR-4800-3p | 0.508411 | 0.078461 | -2.69594 | 3.31E-20 | 1.48E-19 |
| hsa-miR-144-5p | 922.6715 | 119.5941 | -2.94767 | 1.89E-35 | 3.02E-34 |
| hsa-miR-3154 | 0.844047 | 0.092414 | -3.19113 | 2.09E-30 | 1.91E-29 |
| hsa-miR-139-3p | 98.1043 | 9.20227 | -3.41425 | 2.12E-47 | 1.39E-45 |
| hsa-miR-6788-3p | 1.140055 | 0.092585 | -3.62218 | 7.84E-22 | 3.88E-21 |
| hsa-miR-486-5p | 2034.995 | 151.3574 | -3.74899 | 1.26E-47 | 9.22E-46 |
| hsa-miR-4732-3p | 6.142497 | 0.398508 | -3.94614 | 6.96E-43 | 2.54E-41 |

**Supplementary Table 5. miRNAs associated with NSCLC prognosis identified through ROC curve analysis**

| miRNA | AUC |
| --- | --- |
| hsa-miR-4664-3p | 0.565925486708481 |
| hsa-miR-548v | 0.413671493443263 |
| hsa-miR-548d-3p | 0.545874895384512 |
| hsa-miR-556-3p | 0.443551431845162 |
| hsa-miR-195-5p | 0.426099665701431 |


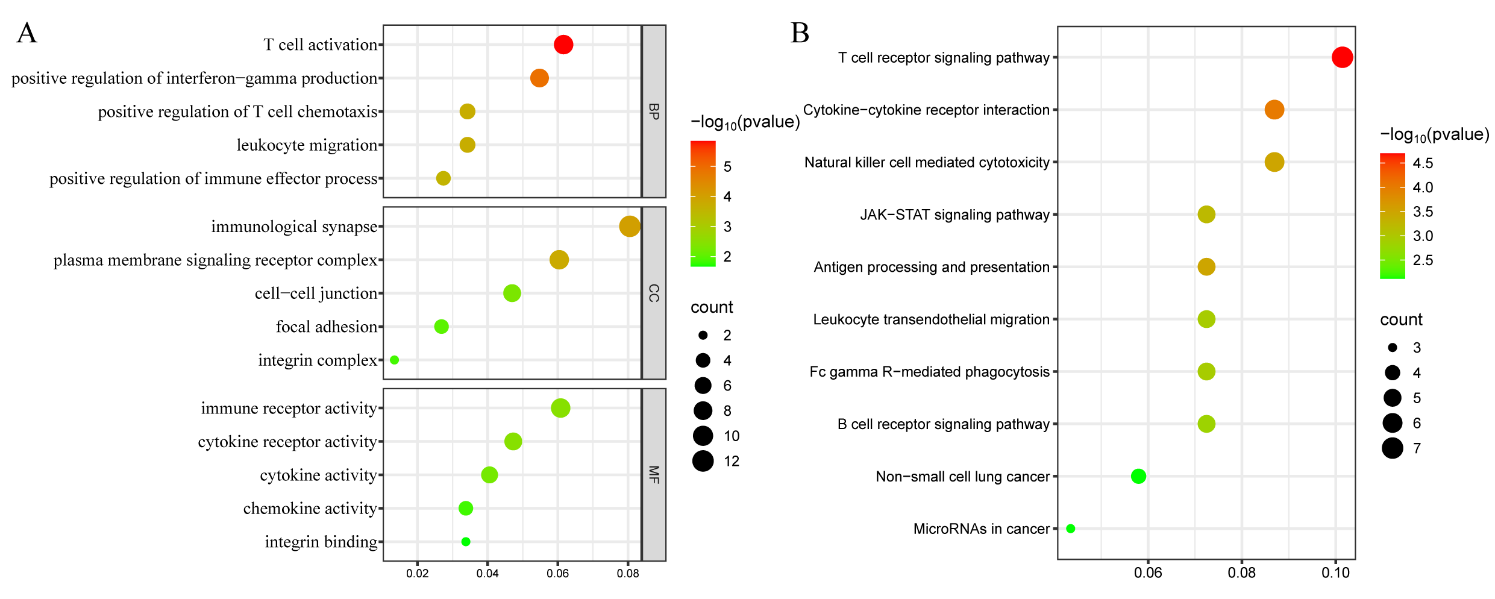


**Supplementary Figure 1.** Gene enrichment analysis of potential target genes of miR-4664-3p

**
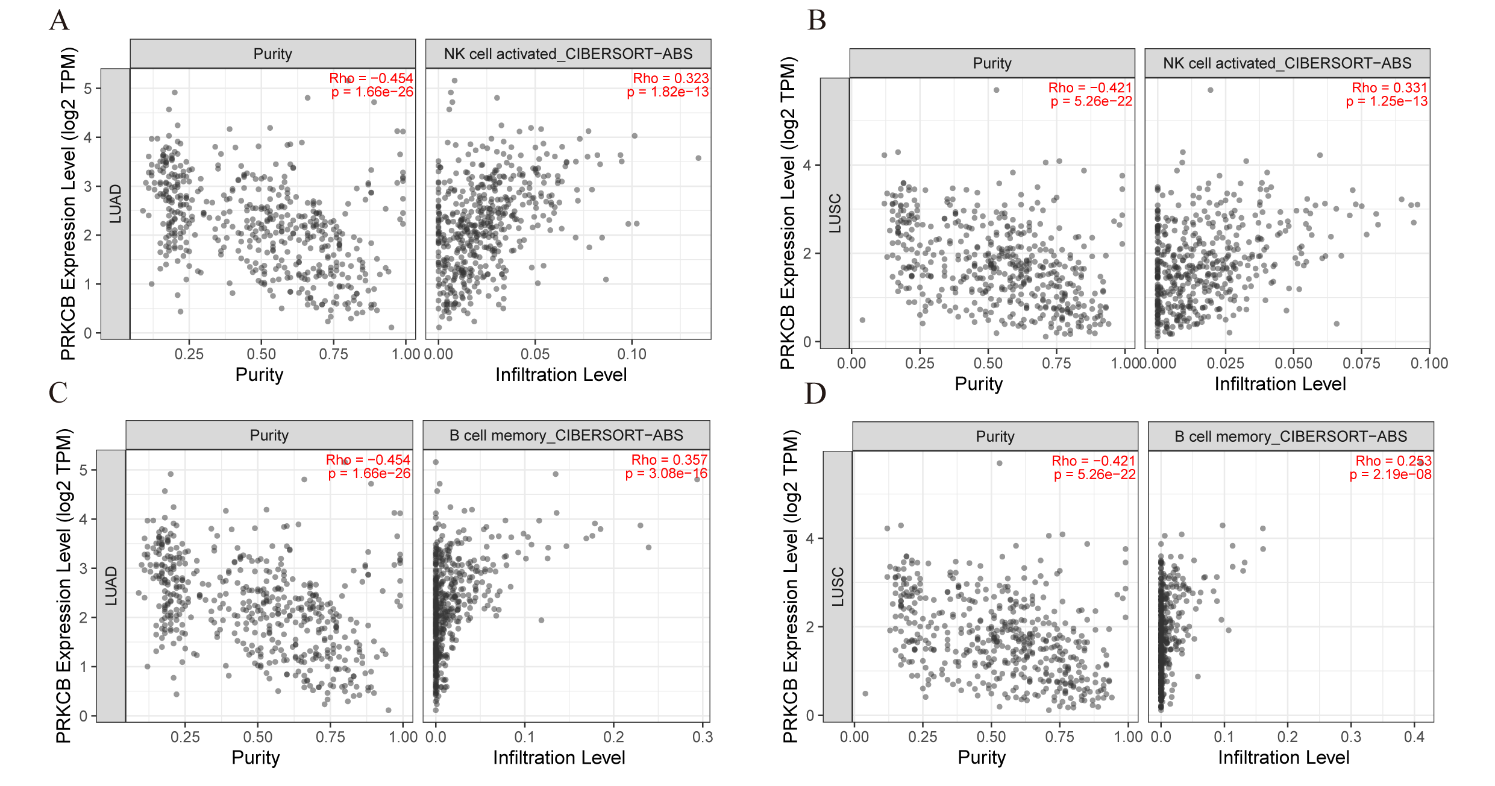
**

**Supplementary Figure 2.** Correlation of PRKCB expression with immune cell infiltration in LUAD and LUSC


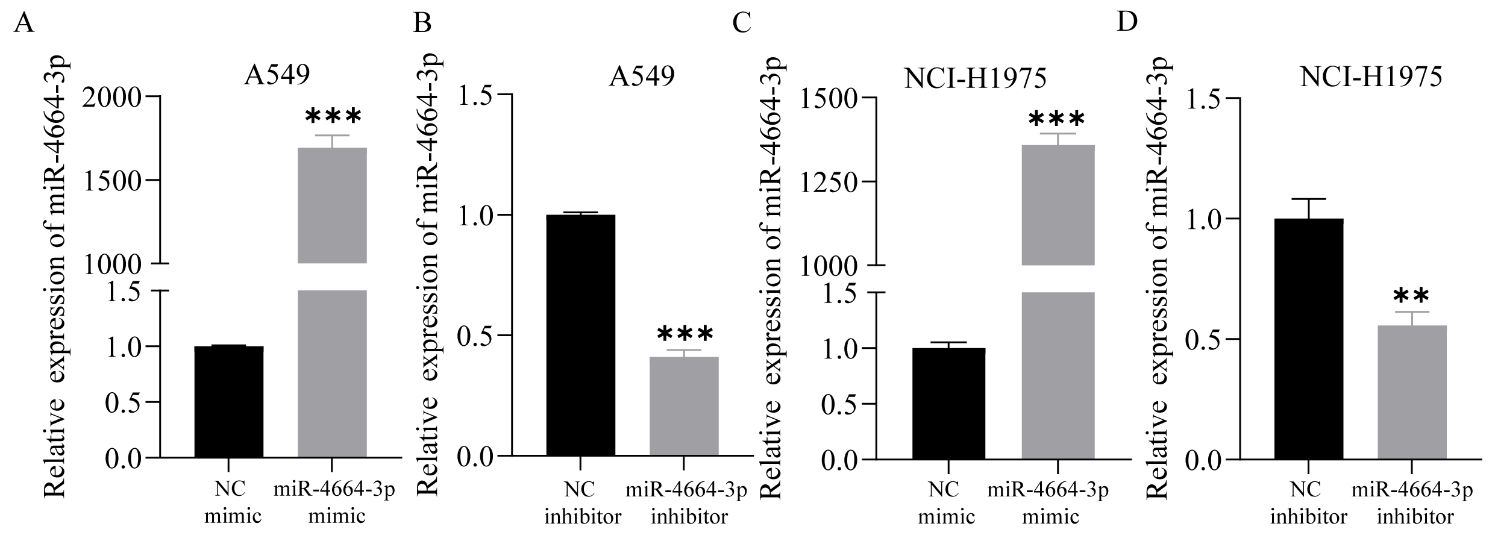


**Supplementary Figure 3.** qRT-PCR analysis of miR-4664-3p expression in transfected cells


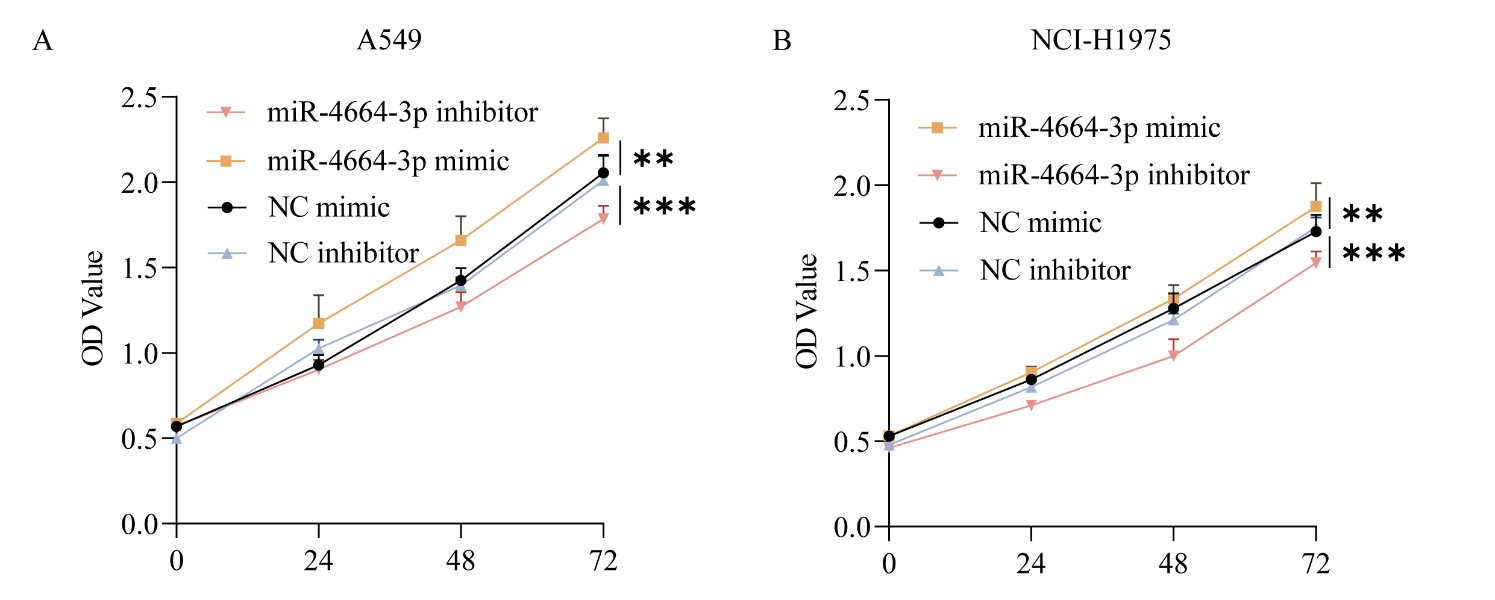


**Supplementary Figure 4.** CCK-8 assay to assess cell viability in A549 and NCI-H1975 cells
